# SOAPfuse: an algorithm for identifying fusion transcripts from paired-end RNA-Seq data

**DOI:** 10.1186/gb-2013-14-2-r12

**Published:** 2013-02-14

**Authors:** Wenlong Jia, Kunlong Qiu, Minghui He, Pengfei Song, Quan Zhou, Feng Zhou, Yuan Yu, Dandan Zhu, Michael L Nickerson, Shengqing Wan, Xiangke Liao, Xiaoqian Zhu, Shaoliang Peng, Yingrui Li, Jun Wang, Guangwu Guo

**Affiliations:** 1 BGI Tech Solutions Co., Ltd, Beishan Industrial Zone, Yantian District, Shenzhen 518083, China; 2 BGI-Shenzhen, Beishan Industrial Zone, Yantian District, Shenzhen 518083, China; 3 School of Life Science and Technology, University of Electronic Science and Technology of China, No.4, Section 2, North Jianshe Road, Chengdu 610054, China; 4 School of Bioscience and Bioengineering, South China University of Technology, Guangzhou Higher Education Mega Centre, Panyu District, Guangzhou 510006, China; 5 Cancer and Inflammation Program, National Cancer Institute, National Institutes of Health, 1050 Boyles Street, Frederick, MD 21702, USA; 6 School of Computer Science, National University of Defense Technology, No.47, Yanwachi street, Kaifu District, Changsha, Hunan 410073, China; 7 State Key Laboratory of High Performance Computing, National University of Defense Technology, No.47, Yanwachi street, Kaifu District, Changsha, Hunan 410073, China; 8 The Novo Nordisk Foundation Center for Basic Metabolic Research, University of Copenhagen, DK-1165 Copenhagen, Denmark; 9 Department of Biology, University of Copenhagen, DK-1165 Copenhagen, Denmark

## Abstract

We have developed a new method, SOAPfuse, to identify fusion transcripts from paired-end RNA-Seq data. SOAPfuse applies an improved partial exhaustion algorithm to construct a library of fusion junction sequences, which can be used to efficiently identify fusion events, and employs a series of filters to nominate high-confidence fusion transcripts. Compared with other released tools, SOAPfuse achieves higher detection efficiency and consumed less computing resources. We applied SOAPfuse to RNA-Seq data from two bladder cancer cell lines, and confirmed 15 fusion transcripts, including several novel events common to both cell lines. SOAPfuse is available at http://soap.genomics.org.cn/soapfuse.html.

## Background

Gene fusions, arising from the juxtaposition of two distinct regions in chromosomes, play important roles in carcinogenesis and can serve as valuable diagnostic and therapeutic targets in cancer. Aberrant gene fusions have been widely described in malignant hematological disorders and sarcomas [1-3], with the recurrent *BCR*-*ABL *fusion gene in chronic myeloid leukemia as the classic example [4]. In contrast, the biological and clinical impact of gene fusions in more common solid tumor types has been less appreciated [2]. However, recent discoveries of recurrent gene fusions, such as *TMPRSS2*-*ERG *in a majority of prostate cancers [5,6], *EML4*-*ALK *in non-small-cell lung cancer [7] and *VTI1A*-*TCF7L2 *in colorectal cancer [8], point to their functionally important role in solid tumors. These fusion events were not detected until recently due to technical and analytic problems encountered in the identification of balanced chromosomal aberrations in complex karyotypic profiles of solid tumors.

Massively parallel RNA sequencing (RNA-Seq) using a next-generation sequencing (NGS) platform provides a revolutionary, new tool for precise measurement of levels of transcript abundance and structure in a large variety of species [9-16]. In addition, RNA-Seq has been proven to be a sensitive and efficient approach to gene fusion discovery in many types of cancers [17-20]. Compared with whole genome sequencing, which is also able to detect gene-fusion-creating rearrangements, RNA-Seq identifies fusion events that generate aberrant transcripts that are more likely to be functional or causal in biological or disease settings.

Recently, several computational methods, including FusionSeq [21], deFuse [22], TopHat-Fusion [23], FusionHunter [24], SnowShoes-FTD [25], chimerascan [26] and FusionMap [27], have been developed to identify fusion transcript candidates by analyzing RNA-Seq data. Although these methods were capable of detecting genuine fusion transcripts, many challenges and limitations remain. For example, to determine the junction sites in a given fusion transcript, FusionSeq selected all exons that were potentially involved in the junction from both of the gene pairs, and then covered the exons with a set of 'tiles' that were spaced one nucleotide apart [21]. A fusion junction library was constructed by creating all pairwise junctions between these tiles, and the junctions were identified by mapping the RNA-Seq reads to the junction library. This module makes FusionSeq time consuming, especially for genes with more and larger exons. In addition, FusionHunter identified only fusion transcripts with junction sites at the exon edge (splicing junction), but could not detect a fusion transcript with junction sites in the middle of an exon [24]. Many homologous genes and repetitive sequences often masquerade as fusion events due to ambiguous alignments of short NGS sequencing reads. The lack of effective filtering mechanisms promoted frequent detection of spurious fusion transcripts. Furthermore, several software consumed large amounts of computational resources (CPU time and memory usage), which was a serious problem when analyzing hundreds of samples in parallel.

**Figure 1:**
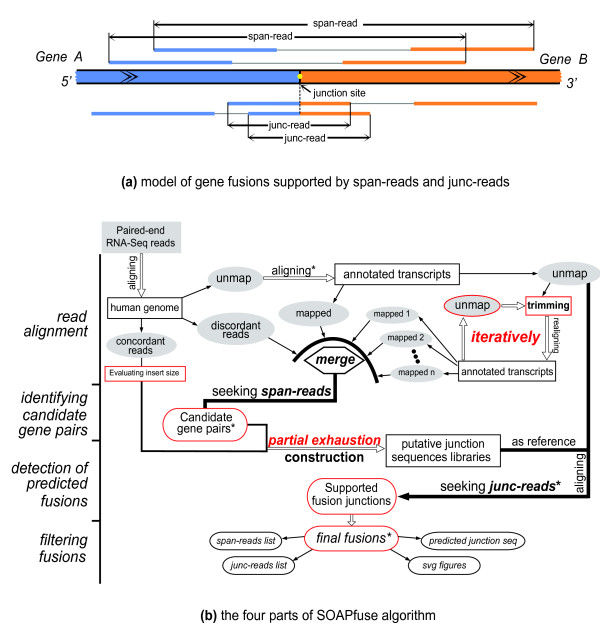
**Framework of SOAPfuse for discovering fusion events**. (a) Model of the fusion event from *Gene A *and *Gene B *that is supported by span-reads and junc-reads. *Gene A *and reads mapped to it are in blue, and *Gene B *is in orange. The junction site is marked by a yellow dot. Two span-reads and two junc-reads are shown. **(b) **The four parts of the SOAPfuse algorithm: read alignment against the human genome reference and annotated transcript sequences; identifying candidate gene pairs by seeking span-reads; detection of predicted fusions; filtering fusions with several criteria to generate the final high-confidence fusion transcript list. Methods with central roles in the algorithm are indicated in red. Steps marked by an asterisk indicate key filtering steps.

To address the limitations above, we present a new algorithm, SOAPfuse, which detects fusion transcripts in cancer from paired-end RNA-Seq data. SOAPfuse combines alignment of RNA-Seq paired-end reads against the human genome reference sequence and annotated genes, with detection of candidate fusion events. It seeks two types of reads supporting a fusion event (Figure 1a): discordant mapping paired-end reads (span-read) that connect the candidate fusion gene pairs; and junction reads (junc-read) that confirm the exact junction sites. SOAPfuse applies an improved partial exhaustion algorithm to efficiently construct a putative junction library and also adopts a series of filters and quality control measures to discriminate likely genuine fusions from sequencing and alignment artifacts (Figure 1b; see Materials and methods). The program reports a high-confidence list of fusion transcripts with the precise locations of junction sites at single nucleotide resolution. Furthermore, SOAPfuse supplies the predicted junction sequences of fusion transcripts, which are helpful for the design of bilateral primers in preparation for RT-PCR validation. Moreover, SOAPfuse creates schematic diagrams that can display the alignment of supporting reads (span-reads and junc-reads) on junction sequences and expression levels of exons from each gene pair. Figures are created in lossless image format (SVG, scalable vector graphics) and, with detailed information on fusion events, will facilitate comprehensive characterization of fusion transcripts at single base resolution and will greatly aid manual selection of the fusion events of interest for further research. SOAPfuse can distinguish specific features of RNA-Seq data, such as insert size and read length, so it still works well even when a single sample includes different types of paired-end RNA-Seq data.

## Results

### Evaluation of performance and sensitivity of SOAPfuse

**Figure 2:**
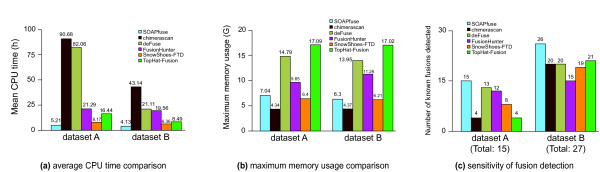
**Performance and sensitivity comparison among six tools based on datasets from previous studies**. Dataset A is from a melanoma study, and dataset B is from breast cancer research. **(a-c) **Average CPU time (a), maximum memory usage (b) and sensitivity of fusion detection (c) of six tools are shown in histograms with detailed values (top).

To assess the performance and sensitivity of SOAPfuse, we applied SOAPfuse to paired-end RNA-Seq datasets from two previous studies: dataset A, consisting of six melanoma samples and one chronic myelogenous leukemia sample, in which 15 confirmed fusions were detected [19]; and dataset B from four breast cancer cell lines with 27 validated fusions [20]. According to Sanger sequences, we characterized these fusion transcripts using release 59 of the Ensembl annotation database [28], including gene symbols, chromosome locations and exact genomic coordinates of junction sites (Additional file 1). To compare SOAPfuse with other published software (Additional file 2), we also run deFuse [22], TopHat-Fusion [23], FusionHunter [24], SnowShoes-FTD [25] and chimerascan [26] on both RNA-Seq datasets. FusionSeq [21] and FusionMap [27] were abandoned due to computational limitations (Additional file 3). We examined different parameters for each tool to obtain higher sensitivity with lower consumption of computational resources (Additional file 3). For a given fusion event, the distance between a junction site identified by these tools and the real one as determined by previous reports should be less than 10 bp, or the fusion event was considered as not detected. Figure 2 shows the computing resources (CPU time and memory usage) and sensitivity for SOAPfuse and the other five methods (Additional file 4).

For dataset A, which contains approximately 111 million paired-end reads, SOAPfuse consumed the least CPU time (approximately 5.2 hours) and the second least memory (approximately 7.1 Gigabytes) to complete the data analysis (including the alignment of reads against reference), and was able to detect all 15 fusion events. DeFuse and FusionHunter detected comparable numbers of known fusion events (12 to 13 of the 15 fusions), but took 82.1 and 21.3 CUP hours, respectively, at least four times as much as SOAPfuse (Additional file 5). The computational resource cost of SnowShoes-FTD was comparable with SOAPfuse, but SnowShoes-FTD identified only 8 of 15 events. The remaining two tools, chimerascan and TopHat-Fusion, detected four confirmed fusion events but used significantly more CPU hours or memory usage. For dataset B containing approximately 55 million paired-end reads, SOAPfuse detected 26 of the 27 reported fusion events with 4.1 CPU hours and 6.3 Gigabytes memory. The other five tools were able to identify comparable numbers of reported fusions (15 to 21) and cost at least 6.4 hours CPU time. One fusion event, *NFS1*-*PREX1*, was missed by all methods, including SOAPfuse (Additional file 3).

The process of data analysis for all six tools included two stages: read alignment then detection of fusion events. For both datasets, SOAPfuse, SnowShoes-FTD, and chimerascan consumed less memory than the other three tools. Chimerascan used less memory than SOAPfuse because it used Bowtie [29], which required less memory than SOAP2 [30] in SOAPfuse, to align reads. The memory usage of the other tools (deFuse, FusionHunter, and TopHat-Fusion) were almost two to three times that of SOAPfuse. They reached maximum memory usage at the fusion detection stage, but not at the read alignment stage, which suggests there may still be room for algorithm improvement for fusion detection. SOAPfuse uses several optimized algorithms to reduce memory consumption with low cost to computation speed. For the two datasets, SOAPfuse expended less CPU time and memory than most of the other five tools, and reached the highest detection sensitivity, with almost all reported fusion events rediscovered (41 of 42), showing its superior performance and high sensitivity.

### Estimate of the false negative and false positive rates by simulated datasets

**Figure 3:**
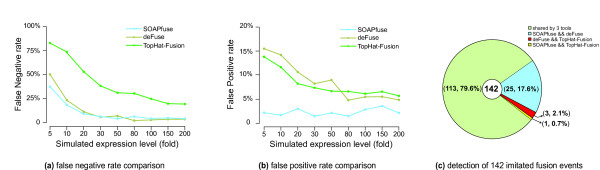
**Evaluation of false negative and false positive rates of SOAPfuse based on simulated datasets**. A comparison among three tools based on simulated fusion events is shown with different expression levels of the fusion transcripts. **(a,b) **FN rate (a) and FP rate (b) of three tools are shown in line graphs. **(c) **Distribution of 142 simulated fusion events detected by the three methods. SOAPfuse missed three simulated fusion events (red) that were identified by both deFuse and TopHat-Fusion.

To estimate the false negative (FN) and false positive (FP) rates of fusion detection by SOAPfuse, we applied SOAPfuse to a simulated RNA-Seq dataset. We used the short-read simulator provided by MAQ [31] to generate paired-end RNA-Seq reads from 150 simulated fusions with nine different expression levels (5- to 200-fold; Additional files 3 and 6). We mixed simulated reads with the RNA-Seq dataset (approximately 19 million paired-end reads) from human embryonic stem cells, which was also used as background data by FusionMap [27]. Chimerascan, FusionHunter and SnowShoes-FTD only detected fusion events with junction sites at the exon boundaries. Their performances could not be evaluated because some simulated fusion events harbored junction sites in the middle of exons. We tested deFuse, TopHat-Fusion and SOAPfuse on simulated paired-end reads. Several strategies were applied to fairly compare the performance of these tools (Additional file 3). In total, 149 (99%) of the 150 fusion events were rediscovered, and 142 (94%) were detected by at least two tools, indicating our simulation was reasonable. To be conservative, the performance comparison was based on the 142 events that were supported by at least two algorithms (Figure 3; Additional file 7).

As expected, FN rates decreased with increasing expression levels of fusion transcripts (Figure 3a). SOAPfuse and deFuse achieved the lowest FN rates at 5% with fusion transcript expression levels of 30-fold or greater. TopHat-Fusion had higher FN rates, especially at low fusion transcript expression levels (5- to 20-fold). For the FP rate (Figure 3b), only SOAPfuse achieved <5% at different fusion transcript expression levels, while deFuse and TopHat-Fusion had higher FP rates at lower fusion transcript expression levels.

Generally, lower FN rates and lower FP rates are contradictory for detection of fusions; however, SOAPfuse and deFuse are good at reducing FN and FP rates during fusion transcript identification. SOAPfuse missed three simulated fusions, which are detected by both deFuse and TopHat-Fusion (Figure 3c), revealing a weakness in analysis of homologous gene sequences and short fusion transcripts of long genes (Additional file 3). In summary, SOAPfuse showed optimal performance with low FN and FP rates at different expression levels of fusion transcripts.

### Application to bladder cancer cell lines

**Figure 4:**
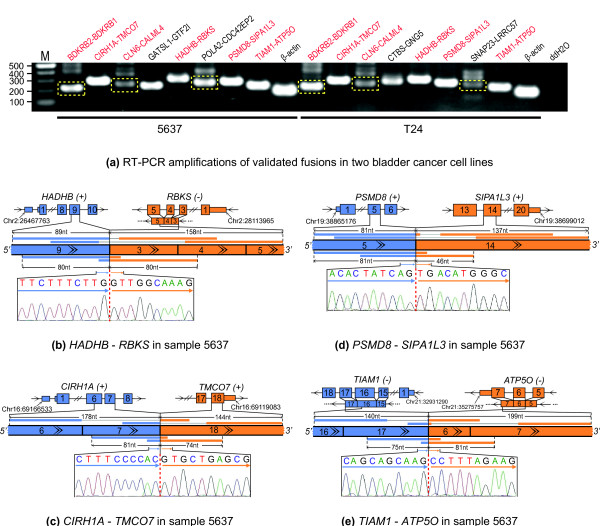
**Confirmed fusions in two bladder cancer cell lines**. (a) RT-PCR amplifications of confirmed fusions in two bladder cancer cell lines. Marker (M), positive (β-actin) and negative (ddH_2_O) controls are also shown. Fusion events in red are detected in both cell lines. For fusions that have multiple RT-PCR products, genuine amplicons of a fusion transcript are boxed in yellow. One fusion, *SNAP23-LRRC57*, reported by deFuse, is further discussed in the text. **(b-e) **Fusion events that indicate potential chromosomal rearrangements, including potential inversion (b) and intrachromosomal translocations (c-e), are shown. Blue segments are upstream genes, and downstream genes are in orange. Gene symbols are followed with their DNA strands. Exons around the junction sites are drawn with a double slash indicating exons that are not shown. The start positions of upstream genes and end positions of downstream genes are noted with a colon separating chromosomal location and reference genome coordinate. The span-reads and junc-reads from RNA-Seq are shown over and under the junction sequences, respectively. Sanger sequencing of junction sequences are displayed under the junction sites.

We next applied SOAPfuse to two bladder cancer cell lines, 5637 and T24. We performed high-throughput RNA-Seq, using Illumina HiSeq sequencing technology, on mRNA from both cell lines and acquired more than 30 million paired-end reads for each (Table 1; see Materials and methods). SOAPfuse identified a total of 16 fusion transcripts, all of which are intrachromosomal and fused at the exon boundaries. We designed primers for RT-PCR experimental validation of all predicted fusions, and Sanger sequencing of the amplicons confirmed 15 (94%) events, of which 6 were detected in both cell lines (Figure 4a; Table 2; Additional file 8). Detailed analysis showed that several confirmed fusion events (Table 2) might be consequences of chromosomal rearrangements. For example, the *HADHB-RBKS *fusion transcript (Figure 4b) fuses two genes from different DNA strands, indicating a potential inversion (Figure S1a in Additional file 9). Furthermore, some fusions implied possible intrachromosomal translocations (Figure S1b in Additional file 9), such as *CIRH1A-TMCO7, PSMD8-SIPA1L3*, and *TIAM1-ATP5O *(Figure 4c-e). Intrachromosomal translocations as a mechanism to create fusions were also found in ovarian carcinoma [32] and glioblastoma [33]. To our knowledge, all the confirmed fusion events have not been reported by previous studies on bladder cancer, indicating their potential significance for further research.

**Table 1:** RNA-Seq data from two bladder cancer cell lines

Sample ID	Read type	Insert size	Read length	Number of paired-end reads
5637	Paired end	200	90	32,228,742
T24	Paired end	200	90	36,830,100

**Table 2:** Confirmed fusion events from two bladder cancer cell lines

Sample ID	Fusion genes (5'-3')	Chromosome (5'-3')	5' position	3' position	Fusion reads (span/junc)	Detected in both cell lines	Potential chromosomal rearrangement
5637	*BDKRB2-BDKRB1*	14-14	96703518	96728989	2/3	Yes	No
5637	*CIRH1A-TMCO7*	16-16	69184807	69117388	20/20	Yes	Yes
5637	*CLN6-CALML4*	15-15	68521840	68489966	4/4	Yes	No
5637	*GATSL1-GTF2I*	7-7	74867229	74143124	3/15	No	Yes
5637	*HADHB-RBKS*	2-2	26502983	28070964	12/10	Yes	Yes
5637	*POLA2-CDC42EP2*	11-11	65063461	65088015	3/5	No	No
5637	*PSMD8-SIPA1L3*	19-19	38871639	38673159	5/5	Yes	Yes
5637	*TIAM1-ATP5O*	21-21	32537279	35276325	9/21	Yes	Yes
T24	*BDKRB2-BDKRB1*	14-14	96703518	96728989	3/3	Yes	No
T24	*CIRH1A-TMCO7*	16-16	69184807	69117388	19/24	Yes	Yes
T24	*CLN6-CALML4*	15-15	68521840	68489966	6/5	Yes	No
T24	*CTBS-GNG5*	1-1	85028940	84967653	3/6	No	No
T24	*HADHB-RBKS*	2-2	26502983	28070964	6/7	Yes	Yes
T24	*PSMD8-SIPA1L3*	19-19	38871639	38673159	6/5	Yes	Yes
T24	*TIAM1-ATP5O*	21-21	32537279	35276325	8/28	Yes	Yes

All information is based on release 59 of the Ensembl hg19 annotation database. Six fusions were detected in both bladder cell lines, and five events may be derived from potential chromosomal rearrangements on the genome. For Fusion reads (span/junc), numbers of span-reads and junc-reads are separated by a slash.

**Figure 5:**
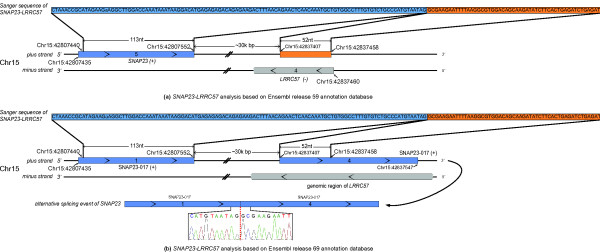
**Analysis of *SNAP23-LRRC57 *reported by deFuse**. (a) *SNAP23-LRRC57 *analysis based on release 59 of the Ensembl annotation database. Sanger sequencing of RT-PCR amplicons of the *SNAP23-LRRC57 *fusion event reported by deFuse is shown. The upstream gene (*LRRC57*) is in blue, and the downstream part is in orange. Gene symbols are followed with their DNA strands. The downstream fusion part is the antisense strand sequence of exon 4 of *LRRC57*. **(b) **In the latest release (release 69) of the Ensembl annotation database, the downstream part of the *SNAP23-LRRC57 *fusion is annotated as part of exon 4 of SNAP23-017, one of the *SNAP23 *transcripts. The fusion *SNAP23-LRRC57 *reported by deFuse is in fact an alternative splicing event in gene *SNAP23*.

We also used deFuse to reanalyze this dataset and identified 11 fusions, of which 10 (91%) events were able to be confirmed by RT-PCR experiments. Nine of the ten confirmed events were also detected by SOAPfuse (Table S10 in Additional file 8) and the remaining fusion transcript (*SNAP23-LRRC57*; Figure 4a) was missed by SOAPfuse. Sanger sequencing shows that exon 5 of *SNAP23 *is fused to the antisense sequence of exon 4 of *LRRC57*. This implies that deFuse has a somewhat different definition of a fusion compared to SOAPfuse (Figure 5a; Additional file 3). The distance between the junction sites in *SNAP23 *and *LRRC57 *is approximately 30 kbp, which is always allowed by the alternative splicing. We speculated the fusion predicted by deFuse might be an alternative splicing event in the upstream gene, *SNAP23*. So we checked the latest version of the Ensembl annotation database (release 69) and found a transcript sequence (SNAP23-017) of gene *SNAP23 *in which the antisense sequences of exon 4 in *LRRC57 *has been annotated as a new exon in the *SNAP23 *gene (Figure 5b). Based on this discovery, we believe the *SNAP23-LRRC57 *fusion event reported by deFuse is an alternative splicing event in *SNAP23*.

## Discussion

We have developed a new method called SOAPfuse to aid in fusion transcript discovery from paired-end RNA-Seq data. Comparing SOAPfuse with other tools on two previously published datasets, one simulated dataset and two bladder cancer cell line datasets, we authenticated superior performance and high sensitivity of SOAPfuse. By evaluating the program on a simulated dataset, SOAPfuse showed a low FP rate (5%) at different expression levels of fusion transcripts and it also achieved a low FN rate of 5% when the expression levels of fusion transcripts were greater than 30-fold. Using the bladder cancer cell line datasets, we demonstrated with RT-PCR-validated fusions that SOAPfuse has substantially high accuracy (15 of 16, 94%) and we also identified several novel fusion transcripts that may be derived from chromosomal rearrangements.

In the simulated dataset, SOAPfuse missed three fusion transcripts. The program had some difficulties detecting fusion transcripts from gene pairs having highly similar sequences, and fusion transcripts involving short transcripts of long genes. However, preliminary solutions have been applied to remedy these shortcomings successfully (Additional file 3), and will be included in future versions of SOAPfuse. After analyzing the characteristics of the fusion events, we found that several novel fusion transcripts detected in the bladder cancer cell lines were more likely to be derived from chromosomal rearrangements of the DNA. Whole genome sequencing will be helpful for determining whether the fusion transcripts are from genomic DNA variations and if the breakpoints can be detected. We have started to develop a new algorithm to detect chromosomal rearrangements that can generate predicted fusion transcripts from whole genome sequencing data based on the results from SOAPfuse. It will be complementary to SOAPfuse for performing genome analysis of fusions with tools like CREST [34]. We will continuously refine SOAPfuse and update it on our official website.

## Conclusions

Here we present an optimized publicly available methodology for identifying novel fusion transcripts from RNA-Seq data. Our results suggest that SOAPfuse achieves better performance than other published tools and it produces a highly accurate list of fusion events in a time-efficient manner. Furthermore, it provides predicted junction sequences and schematic diagrams of fusion events, which are helpful to analyze detected fusions. Overall, SOAPfuse is a useful method that will enable other research groups to make discoveries from their own RNA-Seq data collections.

## Materials and methods

### Outline of the general approach

**Figure 6:**
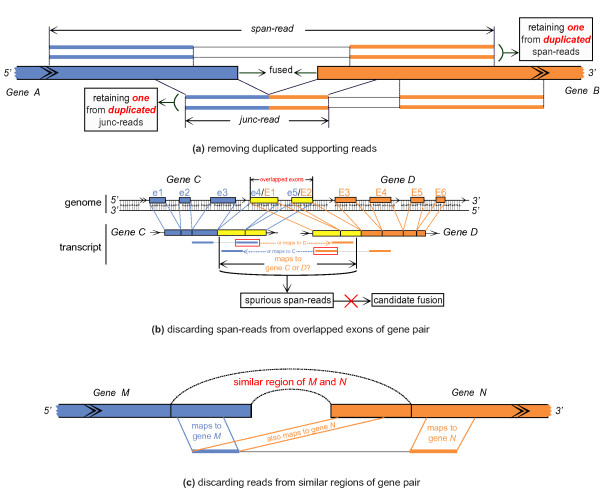
**Basic filtering of candidate gene pairs in SOAPfuse**. (a) Duplicated span-reads and junc-reads are removed before calculating the number of supporting reads and only one duplicated read is retained. **(b) ***Genes C *and *D *are adjacent, and they share two exons: exon 4 and exon 5 from *Gene C *overlap with exon 1 and exon 2 of *Gene D*, respectively. Span-reads from the overlapped exons are excluded by SOAPfuse. **(c) **Gene pair M and N has regions with homogenous/similar sequences and reads from these regions are filtered out.

SOAPfuse seeks two types of reads (span-reads and junc-reads; Figure 1a) to identify fusion transcripts. Paired-end reads that map to two different genes (a gene pair) are defined as span-reads, and reads covering the junction sites are called junc-reads. Span-reads are used to identify candidate gene pairs, and junc-reads are used to characterize the exact junction sites at single base resolution. Duplicate span-reads and junc-reads are removed before calculating the number of supporting reads (Figure 6a). SOAPfuse contains nine steps in its pipeline (Additional file 10), and can be divided into four parts (Figure 1b): (i) read alignment (steps S01 to S03); (ii) identifying candidate gene pairs (steps S04 and S05); (iii) detection of predicted fusions (steps S06 and S07); and (iv) filtering fusions (steps S08 and S09). A detailed description of the algorithm is in Additional file 3.

### Read alignment

SOAPfuse initially aligns paired-end reads against the human reference genome sequence (hg19) using SOAP2 [30] (SOAP-2.21; step S01 in Additional file 10). We divided the reads into three types according to the read alignment results: PE-S01, SE-S01 and UM-S01, where PE stands for paired-end mapped result, SE for single-end mapped result, and UM for unmapped read. PE-S01 reads indicate the paired-end reads mapping to the genome with the proper insert sizes (<10,000 bp). SE-S01 contains paired-end reads in which only one of two ends mapped to the reference genome, and paired-end reads indicating a fragment with an abnormal insert size or mapped orientation. All unmapped reads are saved in UM-S01 with a FASTA format. PE-S01 is used to evaluate insert size (Additional file 3). SOAPfuse then aligns UM-S01 reads against annotated transcripts (Ensembl release; step S02 in Additional file 10) and generates SE-S02 and UM-S02. To filter unmapped reads caused by small indels, UM-S02 reads are realigned to annotated transcripts using BWA [35] (BWA-0.5.9; maximum number of gap extensions is 5), and the remaining unmapped reads are called filtered-unmapped (FUM).

### Iteratively trimming and realigning reads

**Figure 7:**
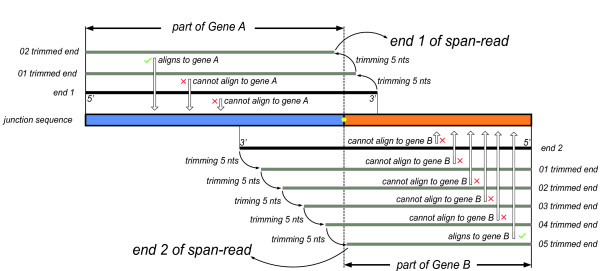
**Trimming and realigning the paired-end reads in which both 3' ends overlap each other**. A junction sequence is shown with the junction site noted by a yellow dot. The blue region is from *Gene A*, and orange is from *Gene B*. The paired-end read with overlapped 3' ends (black thick line) cannot map to *Gene A *and *Gene B*, as reads cover the junction site. A series of trimmed reads (gray thick line) are obtained by iteratively trimming 5 nucleotides (nts) each time from the 3' ends until the reads could map to *Gene A *and *Gene B*. In this example, end 1 of a paired-end read requires two cycles of trimming to achieve successful alignment, while end 2 needs five cycles.

The latest protocols for NGS RNA-Seq library preparation can generate paired-end reads with an insert size shorter than the total length of both reads (with the 3' ends of both reads overlapped). The paired-end reads with overlapped 3' ends may come from the junction regions containing the junction sites and these paired-end reads are not mapped to the reference if the overlapped regions cover the junction sites. These reads are components of FUM generated in step S02 (Additional file 10) and cannot become span-reads, which will reduce the capability of fusion detection. SOAPfuse estimates whether the number of these paired-end reads with overlapped 3' ends exceeds the threshold (20% of total reads by default). If yes, or the user enables a trimming operation accessible in the configuration file, SOAPfuse will iteratively trim and realign FUM reads to annotated transcripts (Figure 7; step S03 in Additional file 10). The length of reads after trimming should be at least 30 nucleotides (default parameter in SOAPfuse). The trimmed reads that are able to be mapped to annotated transcripts are stored in SE-S03 (Additional file 3). Two steps were used to finish the trimming and realigning operation: first, FUM reads were progressively trimmed off five bases from the 3'-end and mapped to annotated transcripts again until a match was found; second, using the same strategy, we trimmed the remaining FUM reads from the 5'-end. All mapped paired-end reads from these two steps were merged together (step S04 in Additional file 10).

### Identifying candidate gene pairs

From all discordantly aligned reads, SOAPfuse seeks span-reads to support candidate gene pairs (step S05 in Additional file 10). Both the span-reads that mapped uniquely to the reference (human genome and annotated transcripts) and the trimmed reads that have multiple hits were used to detect the candidate gene pairs. The maximum hits for each span-read is a parameter in the configuration file. To ensure accurate detection of the fusion gene pairs, SOAPfuse imposes several filters on the predicted candidate gene pair list (Additional file 3), such as excluding gene pairs from the same gene families and pairs with overlapped or homogenous exon regions (Figure 6b).

### Determining the upstream and downstream genes in the fusion events

**Figure 8:**
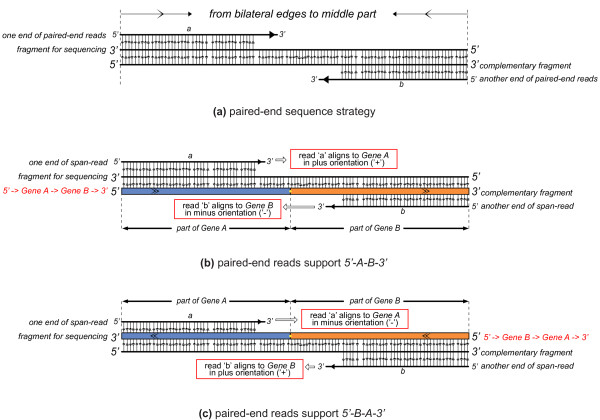
**Determining the upstream and downstream genes in fusion events**. (a) A fragment of paired-end sequencing is shown with its complementary fragment. Paired-end reads (reads 'a' and 'b') are shown with their sequencing direction (from 5' to 3', noted by arrows on reads). Read 'a' is generated from the fragment itself, while read 'b' is from the complementary fragment. The sequencing orientation is from bilateral edges to the middle of the fragment, so the paired-end reads are generated head-to-head. **(b,c) **Different classifications of span-read (read 'a' and 'b') support different upstream and downstream genes. The gene aligned by reads in the plus orientation must be the upstream gene. In (b), read 'a' aligns to *Gene A *in a plus orientation. Based on the paired-end sequencing shown in (a), *Gene A *must be the upstream gene and *Gene B *must be the downstream gene. In (c), read 'b' aligns to *Gene B *in a plus orientation. So, *Gene B *is an upstream gene and *Gene A *is a downstream gene.

After obtaining the candidate gene pairs, the upstream and the downstream genes of the fusion were determined based on the information from span-read alignment against the reference. In the process of paired-end sequencing, the fragments are sequenced from bilateral edges to the middle part: one end starts from the 3' end of the fragment, while the other end starts from the 3' end of the complementary base-pairing sequence of the fragment (Figure 8a). This information is used to define the up- and downstream genes in a fusion transcript.

A span-read (paired-end reads 'a' and 'b') supports a candidate gene pair (*Gene A *and *Gene B*). According to the serial number ('1' or '2') and mapped orientation ('+' or '-') of paired-end reads (read 'a' and 'b'), there are 16 combinations, but only 4 are rational. These four combinations support two types of fusions in which the upstream and downstream genes are different (Additional file 11. Table S12). The judgment rule is: the gene aligned by reads in the plus orientation must be the upstream gene. Here, we presume that read 'a' maps to *Gene A *and read 'b' maps to *Gene B *(Figure 8b,c). In Figure 8b, read 'a' aligns to *Gene A *(annotated transcripts) in the plus orientation, so *Gene A *must be the upstream gene; while in Figure 8c, read 'b' aligns to *Gene B *in the plus orientation, so *Gene B *must be the upstream gene. According to this rule, SOAPfuse defines the upstream and downstream genes in fusion events.

### Obtaining the fused regions

**Figure 9:**
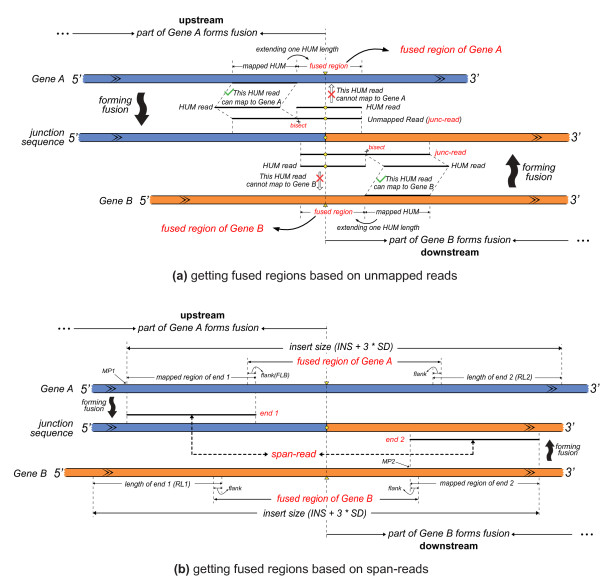
**Obtaining fused regions by two methods**. A junction sequence in a fusion transcript from a gene pair, *Gene A *and *Gene B*, in blue and orange, respectively, is shown. The junction site is displayed as yellow round dots on the fusion sequence. **(a) **Two unmapped reads (candidate junc-reads) are shown around the fusion sequence. Each read is bisected into two isometric HUM reads: one HUM can map to one gene of the pair, while the other one cannot map to the gene as it covers the junction site (yellow round dot). From the location of the mapped HUM read, SOAPfuse extends one HUM read-length to obtain the fused region, in which the junction site is located (yellow triangle). **(b) **Span-read mapping to the gene pair is shown. End 1 maps to *Gene A *(with position *MP1*), and end 2 maps to *Gene B *(with position *MP2*). From the mapped positions of both ends, SOAPfuse determines the potential fused region based on insert sizes (INS), standard deviation of insert sizes (*SD*), and the length of reads (*RL1 *and *RL2 *for both ends, respectively) and extends proper flanking bases to obtain the fused region.

Before we defined the fused regions in which the junction sites may located, we obtained a non-redundant transcript sequence from transcript(s) of each annotated gene (Additional file 3). Two methods were used to define the fused regions in gene pairs. In the first method, SOAPfuse bisects each FUM read, and generates two isometric segments, each called a half-unmapped read (HUM read; step S06 in Additional file 10). HUM reads are aligned against candidate gene pairs with SOAP2. A genuine junction read (junc-read) should have at least one HUM read that does not cover the junction site and could map to one gene of the pair. Based on the mapped HUM read, SOAPfuse extends one HUM read length from the mapped position in non-redundant transcripts to define the fused region wherein the junction site might be located (Figure 9a). For HUM reads with multiple hits, all locations of the hits are taken into account. Original reads of mapped HUM reads are called as useful unmapped reads (UUM read).

SOAPfuse also uses span-reads to detect the fused regions in candidate gene pairs (step S07-a in Additional file 10). Span-reads, the paired-end reads supporting the candidate fusion gene pairs, are derived from the fused transcripts and the junction sites are often located in regions of the fused transcripts between both ends of span-reads. For upstream and downstream genes, we can extend one region with length equal to insert size (evaluated in step S01) from the mapped position of each 3' end span-read to estimate the fused region covering the junction site (Figure 9b). Every gene pair is always supported by at least two span-reads, corresponding to several fused regions that may have overlaps with each other. We presumed that end 1 of a span-read mapped to position *MP1 *in *Gene A*, and end 2 of the span-read mapped to position *MP2 *in *Gene B*. The lengths of ends 1 and 2 of the span-reads are *RL1 *and *RL2*, respectively. The average of insert sizes (*INS*) and their standard deviation (*SD*) are evaluated in step S01. The fused regions were estimated by the following intervals:

The intervals of fused regions for the upstream genes are:

$$\left[MP1+RL1-FLB,\;MP1+INS+3*SD-RL2+FLB-1\right]$$

And the intervals of fused regions for the downstream genes are:

$$\left[MP2+RL2-INS-3*SD+RL1-FLB\;,\;MP2+FLB-1\right]$$

In the above formula, a flanking region with length of *FLB *was considered because sometimes a few bases from the 3' end of a span-read cover the junction sites in the mismatch-allowed alignment.

SOAPfuse combined the fused regions determined by the above two methods to detect the junction sites using the partial exhaustion algorithm as described below.

### Construction of fusion junction sequence library with partial exhaustion algorithm

To simplify the explanation of the algorithm, we call the fused regions determined by the above two methods as fused regions 1 and fused regions 2, respectively. Fused region 1, defined by the mapped HUM reads, is a small region covering the junction sites with length smaller than one NGS read. Fused region 2 is a large region defined by the NGS library insert sizes, which are always much longer than HUM reads. Generally, fused region 1 is more useful than fused region 2 to define the junction sites.

**Figure 10:**
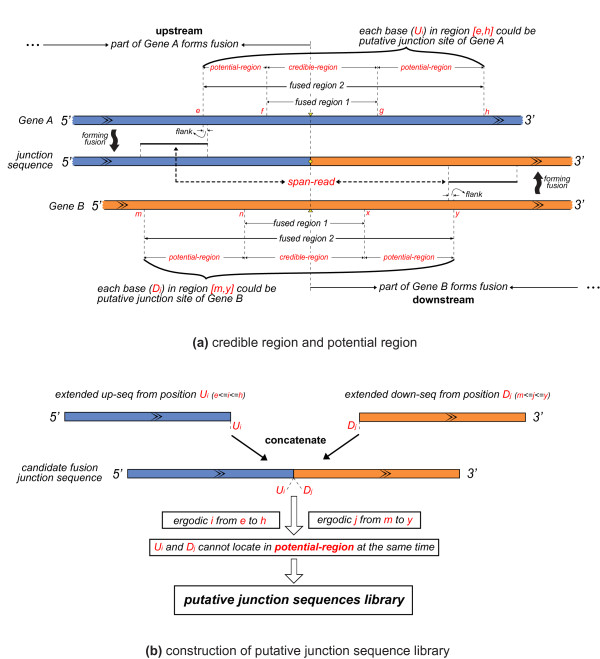
**Building the fusion junction sequence library using a partial exhaustion algorithm**. A junction sequence in a fusion transcript from a gene pair, *Gene A *and *Gene B *in blue and orange, respectively, is shown. The junction site is shown as yellow round dots on the fusion segment, and as yellow triangles on the gene pair. **(a) **Fused regions 1 and 2 from two different methods are shown and fused region 2 is divided into credible-regions and potential-regions with the coordinates of each sub-region labeled in red font. An upstream putative junction site (*U_i_*) is selected from fused region 2 in *Gene A*, and a downstream putative junction site (*D_j_*) is selected from fused region 2 in *Gene B*. **(b) **For each *U_i _*and *D_j_*, SOAPfuse generates the candidate fusion junction sequence by creating pair-wise connections between *U_i _*and *D_j_ . U_i _*and *D_j _*should not be located in potential-regions at the same time.

However, not all mapped HUM reads are from genuine junc-reads. Sometimes, one unmapped read from a given gene does not map this gene as a result of more mismatches than are allowed by SOAP2. Unmapped reads like this are not junc-reads and after the bisection into two HUM reads, one of the HUM reads could be mapped to the original gene, which results in spurious fused regions. Fused region 2 involves alignments of two ends of a span-read simultaneously, which are also filtered by several effective criteria (see the 'Obtaining candidate gene pairs' section). SOAPfuse combined fused regions 1 and 2 to efficiently define the junction sites. SOAPfuse classifies fused region 2 into two types of sub-regions: overlapped parts between fused regions 1 and 2 are called the credible-region, while the other parts of fused region 2 are called the potential-region (Figure 10a).

In order to build the fusion junction sequence library, we covered fused region 2 from each gene pair with 'tiles' that are spaced one nucleotide apart and we finally generated the candidate fusion junction library by creating all pair-wise connections between these tiles (Figure 10b). To eliminate the false positives in the junction sequence library, only the junction sequences in which at least one of two junction sites in a gene pair is located in the credible-region were selected for further analysis. SOAPfuse carried out this partial exhaustion algorithm to reduce the size of the putative junction library and retain genuine junction sequences as much as possible.

### Detection of junction sites in fusion transcripts

To identify the junction sites of fusion transcripts, we mapped the useful-unmapped-reads (UUM reads; see the 'Obtaining the fused regions' section) to the putative fusion junction sequence library to seek the junction reads (step S07-b in Additional file 10). We required that a candidate fusion should be supported by multiple span-reads, junction reads, and other criteria (step S08 in Additional file 10; Additional file 3). To exclude FP fusion events, we removed the initial candidate fusion gene pairs that closed with each other and that had homogenous/overlapping regions around the junction sites (Figure 6c; step S09 in Additional file 10). SOAPfuse not only reports high-confident fusions but also provides the predicted junction sequences for further RT-PCR experimental validations. SVG figures are also created, showing the alignments of supporting reads on junction sequences and expression level of gene pairs (for example, Additional file 12).

### Preparation of simulated datasets

Simulated RNA-Seq data were generated to evaluate the FN and FP rate of SOAPfuse. We generated 150 simulated fusion transcripts in two steps based on human annotated genes. The first step involved randomly selecting candidate gene pairs with several criteria, such as controlling the distance between paired genes and avoiding gene pairs from gene families. The second step involved randomly selecting transcripts and junction sites at the exon edges or in the middle of exons. Using the short-read simulator provided by MAQ [31], we generated paired-end reads at nine sequencing depth (5- to 200-fold) to simulate different expression levels of fusion transcripts. Paired-end reads from H1 human embryonic stem cells were used as background data. Details of the simulation work can be found in Additional file 3.

### Total RNA preparation from bladder cancer cell lines

Two bladder cancer cell lines (5637 and T24) were purchased from the American Type Culture Collection (Manassas, VA, USA). They were cultured in RPMI 1640 medium (Invitrogen, Grand Island, NY, USA) containing 10% fetal bovine serum (Sigma, Saint Louis, MO, USA). Total RNAs were prepared using Trizol (Invitrogen) according to the manufacturer's instructions. They were treated with RNase-free DNase I to remove residual DNA. The quality of total RNAs was evaluated using an Agilent 2100 Bioanalyser.

### cDNA library construction for RNA-Seq

The cDNA libraries were constructed as described in previous studies [36,37]. Briefly, beads (Invitrogen) with oligo (dT) were used to isolate poly (A) mRNA from total RNAs. To avoid priming bias in the process of synthesizing cDNA, mRNA was fragmented before the cDNA synthesis. Purified mRNA was then fragmented in fragmentation buffer at an elevated temperature. Using these short fragments as templates, random hexamer-primers were used to synthesize the first-strand cDNA. The second-strand cDNA was synthesized using buffer, dNTPs, RNase H and DNA polymerase I. Short double-stranded cDNA fragments were purified with a QIAquick PCR extraction kit (Qiagen, Hilden, Germany) and then subjected to an end repair process and the addition of a single 'adenine' base. Next, the short fragments were ligated to Illumina sequencing adaptors. cDNA fragments of a selected size were gel-purified and amplified by PCR. In total, we constructed one paired-end transcriptome library for each cell line, and sequenced them on the Illumina HiSeq2000 platform. Both paired-end libraries were sequenced to a 90-bp read length with insert sizes ranging from 150 to 200 bp. RNA-Seq data from the two bladder cancer cell lines has been submitted to the NCBI Sequence Read Archive (SRA) and are available under accession number [SRA052960].

### Fusion validation by RT-PCR

The digested total RNAs from the bladder cancer cell lines were reverse-transcribed to cDNA for validation using reverse transcriptase (Invitrogen) and oligo-d(t) primers (TaKaRa, Dalian, China). Then, fusion transcripts were validated using RT-PCR amplification followed by Sanger sequencing. For the RT-PCR amplification, the primers were designed using Primer (version 5.0) and all primer sequences can be found in Table S11 in Additional file 8. We carried out the RT-PCR amplifications using TaKaRa Taq™ Hot Start Version and performed reactions in 20 μl volumes with 2 μl of 10× PCR buffer (Mg^2+ ^Plus), 2 μl of dNTP mixture (each 2.5 mM), 2 μl of primers (each 10 μM), 0.5 μl of TaKaRa Taq HS (5 U/μl), 20 ng of cDNA and up to 20 μl using ddH_2_O. The thermocycler program used was the following: (i) 95°C for 4 minutes, (ii) 95°C for 40 seconds, (iii) 55°C to 62°C for 30 seconds, (iv) 72°C for 45 seconds, (v) steps 2 through 4 repeated 35 times, and (vi) 72°C for 10 minutes. The products of RT-PCR amplification were analyzed on a 2% agarose gel to make sure that no unexpected bands were amplified. The purified RT-PCR products were sequenced in forward and reverse directions with the ABI PRISM Big Dye Terminator Cycle Sequencing Ready Reaction kit (version 3) and ABI PRISM 3730 Genetic Analyzer (Applied Biosystems, Foster City, CA, USA). Chromatograms were generated by Chromas (version 2.22), and then were analyzed by BLAT (online genome alignment on the UCSC Genome Browser [38].

## Abbreviations

bp: base pair; CPU: central processing unit; FN: false negative; FP: false positive; FUM: filtered unmapped reads; HUM: half unmapped read bisected from FUM reads; NGS: next-generation sequencing; PE: paired-end mapped result; RL: read length; RT-PCR: reverse transcription polymerase chain reaction; SE: single-end mapped result; SVG: Scalable Vector Graphics; UM: unmapped read.

## Competing interests

The authors declare that they have no competing interests.

## Authors' contributions

JW and GG conceived and designed the basic algorithm of SOAPfuse. WJ and KQ implemented and optimized the algorithm. PS and SW performed the validation experiments. WJ, KQ, MH, PS, QZ and FZ carried out the comparison among different tools. YY developed the cell lines. XL, XZ and SP tested and deployed the software on TianHe series supercomputers. JW, YL and GG supervised the project and gave advice. WJ, KQ, MH, PS, DZ, MLN and GG wrote and revised the manuscript. All authors read and approved the final manuscript.

## Supplementary Material

Additional file 1 Tables S1 - information on all known fusions from two previous studies. Additional detailed information on the known fusions in two previous studies (melanoma and breast cancer researches). All information of fusions is based on release 59 of the Ensembl hg19 annotation database. Click here for file

Additional file 2 **Table S2 - software selected for evaluation of performance and sensitivity**. Click here for file

Additional file 3 Supplementary notes. Click here for file

Additional file 4 **Table S3 - detailed information on performance and fusion detection sensitivity of six tools**. CPU time, maximum memory usage and sensitivity of fusion detection for each tool are shown. For the multiple process operations, CPU time has been translated to single process usage. Click here for file

Additional file 5 **Table S4 - detection screen of six tools on two previous study datasets**. Click here for file

Additional file 6 Tables S5, S6 and S7. Table S5: detailed information on simulated RNA-Seq reads. Table S6: list of 150 simulated fusion events. Table S7: number of fusion-supporting reads for each fusion event. Click here for file

Additional file 7 **Tables S8 and S9**. Table S8: TP and FP rates of SOAPfuse, deFuse and TopHat-Fusion based on simulated datasets. Table S9: detailed information on the simulated fusion events detected by SOAPfuse, deFuse and TopHat-Fusion. Click here for file

Additional file 8 **Tables S10 and S11**. Table S10: fusion transcripts detected by SOAPfuse and deFuse in two bladder cancer cell lines. Table S11: primers and Sanger sequences of confirmed fusions in two bladder cancer cell lines. Click here for file

Additional file 9 Figure S1 - models of fusion transcripts generated by genome rearrangement. (a) Fusion transcript created by genomic inversion of *Gene A *and *Gene B*, which are from different DNA strands. **(b) **Fusion transcript formed by genomic translocation in which *Gene C *and *Gene D *are from the same DNA strand and are far from each other. Click here for file

Additional file 10 Figure S2 - schematic diagrams of nine steps in the SOAPfuse pipeline. The SOAPfuse algorithm consists of nine steps (from S01 to S09) and details of each step are in the Materials and methods or Additional file 3. Click here for file

Additional file 11 Table S12 - sixteen combination of span-read. There are sixteen combinations based on serial numbers of reads and their mapped orientations, but only four combinations are rational, supporting two types of fusions in which the upstream and downstream genes are different. Click here for file

Additional file 12 Figure S3 - schematic diagrams of fusion event *RECK-ALX3*. (a) Alignment of supporting reads against the predicted junction sequence. The upstream part of the junction sequence is in green, and the downstream part is in red. Span-reads are displayed above the predicted junction sequence with the colored dotted line linking paired-end reads. Junc-reads are shown below the junction sequence. **(b,c) **Expression analysis of the exons in *RECK *and *ALX3 *by RNA-Seq read coverage. Transcripts of *RECK *and *ALX3 *are shown below the coordinates. The junction site is shown as a red round dot and a green arrow indicates the transcript orientation in the genome sequence. The region covered by the red line is the region mapped by supporting reads. In this case, we found that the expression levels of *RECK *and *ALX3 *exons at bilateral sides of junction sites are significantly different. The exons involved in the fusion transcript are expressed more highly than other ones. Click here for file
