# Supplementary material for: SOAPfuse: an algorithm for identifying fusion transcripts from paired-end RNA-Seq data
Source: Genome Biol. 2013 Feb 14;14(2):R12. doi: 10.1186/gb-2013-14-2-r12 (PMC4054009; doi:10.1186/gb-2013-14-2-r12)
Supplement: Additional file 3 — Supplementary notes. [file gb-2013-14-2-r12-S3.DOCX]

# Supplementary Notes

**Table of Contents**

[Supplementary Notes 1](#_Toc346870228)

[METHOD OF SOAPFUSE 2](#_Toc346870229)

[Removing duplications from span-reads and junc-reads 2](#_Toc346870230)

[Reads alignment 2](#_Toc346870231)

[Evaluation of insert size of RNA-Seq data 2](#_Toc346870232)

[Trimming and realigning the reads 3](#_Toc346870233)

[Identifying candidate gene pairs 3](#_Toc346870234)

[Determining the upstream and downstream genes in the fusion events 4](#_Toc346870235)

[Getting the non-redundant transcript from multiple transcripts of the gene 4](#_Toc346870236)

[Getting the fused regions 5](#_Toc346870237)

[Construction of fusion junction sequences library with partial exhaustion algorithm 6](#_Toc346870238)

[Detection of junction sites in fusion transcripts 6](#_Toc346870239)

[Classification of fusion transcripts 7](#_Toc346870240)

[EVALUATION OF SOAPFUSE PERFORMANCE 7](#_Toc346870241)

[Introduction 7](#_Toc346870242)

[The reasons for abandoning FusionSeq and FusionMap in comparison 8](#_Toc346870243)

[Criterion for detecting the known fusion events 8](#_Toc346870244)

[Released RNA-Seq data in the first published dataset 8](#_Toc346870245)

[Software Parameters used for two previously published studies 8](#_Toc346870246)

[Parameters for RNA-Seq data from the melanoma research 9](#_Toc346870247)

[Parameters for RNA-Seq data from the breast cancer research 9](#_Toc346870248)

[Fusion transcripts missed by SOAPfuse in the breast cancer data 10](#_Toc346870249)

[Simulating the fusion transcripts 10](#_Toc346870250)

[The first step of fusions simulation 10](#_Toc346870251)

[The second step of fusions simulation 11](#_Toc346870252)

[Simulation of paired end RNA-Seq reads based on the simulated fusion transcripts 11](#_Toc346870253)

[Background data 11](#_Toc346870254)

[Software parameters used for simulated RNA-Seq dataset 11](#_Toc346870255)

[Low standard parameters for low expression level of the fusion transcripts 11](#_Toc346870256)

[Strict software parameters for high expression levels of the fusion transcripts 12](#_Toc346870257)

[Calculation of the false negative (FN) and false positive (FP) rate 12](#_Toc346870258)

[Simulated fusion transcripts missed by SOAPfuse 13](#_Toc346870259)

[Preliminary solutions to simulated events missed by SOAPfuse 14](#_Toc346870260)

[Software parameters used for bladder cancer cell line dataset 14](#_Toc346870261)

[Selecting predicted fusion transcripts to validate by experiment RT-PCR 15](#_Toc346870262)

[WEBSITE 16](#_Toc346870263)

[Official Website 16](#_Toc346870264)

[REFERENCES 17](#_Toc346870265)

# METHOD OF SOAPFUSE

## Removing duplications from span-reads and junc-reads

SOAPfuse seeks two types of reads, span-read and junc-read, to identify fusion transcripts (see Figure 1a in the main text). Paired-end reads that map to any two different genes (gene pairs) are defined as span-reads, and reads covering the junction sites are called as junc-reads. Span-reads are used to identify the candidate gene pairs, and junc-reads are used to detect the junction sites. Different span-reads or junc-reads that mapped to the genome/annotated transcripts with same start and end positions were considered as duplications and only one of the duplications was retained for further analysis (see Figure 6a in the main text).

## Reads alignment

SOAPfuse initially aligns paired-end reads against the human reference genome sequence (hg19) using SOAP2 [[1](#_ENREF_1)] (SOAP-2.21; step S01 in Figure S2). We divided the reads into three types according to the reads alignment results: PE-S01, SE-S01 and UM-S01. PE-S01 reads indicate the paired-end reads mapping to genome with the proper insert sizes (<10,000 bps). SE-S01 includes paired-end reads in which only one of both ends map to reference, and it also includes paired-end reads with the abnormal insert sizes or orientation. All unmapped reads are saved in UM-S01 with a FASTA format. PE-S01 is used to evaluate insert size (see the following section). SOAPfuse then aligns UM-S01 reads against the annotated transcripts (Ensemble release 59th; step S02 in Figure S2) and generates SE-S02 and UM-S02, To filter out unmapped reads caused by small indels, UM-S02 reads is realigned to annotated transcripts using BWA [[2](#_ENREF_2)] (BWA-0.5.9; maximum number of gap extensions is 5), and the remained unmapped reads are called filtered-unmapped (FUM).

## Evaluation of insert size of RNA-Seq data

PE-S01 (step S01 in Figure S2), the paired-end reads concordantly aligned against the human reference genome was used to evaluate insert size of paired-end RNA-Seq library. SOAPfuse is designed to evaluate insert size for each sequencing run of libraries, and users are asked to input precise information on sample ID, library ID, run ID and read length. Based on this information, SOAPfuse can easily distinguish data from different sequencing runs. Paired-end reads that uniquely map to the same exon were selected to evaluate insert size. SOAPfuse calculates the distance between two ends of PE-S01 reads, and evaluates the average of insert sizes (INS) and their standard deviation (SD). The PE-S01 reads with insert size shorter than the threshold (read length + 5) were discarded. Although the length of exons in genes are distributed broadly and may influence the evaluation of insert size, the general insert sizes used in the RNA-Seq library construction, which range from 100nts (nucleotides) to 800nts, can be accurately evaluated. The evaluated insert size is an important parameter for the partial exhaustion algorithm used by SOAPfuse.

To evaluate the influence of the exon length on insert size evaluation, we simulated sequencing datasets (2 x 75nt, paired-end) with different insert sizes based on annotated transcripts. These insert sizes were 100, 200, 300, 400, 500, 600, 700, 800, 900 and 1,000nt, and SD was 20 for each level of insert sizes. One million paired-end reads were simulated by MAQ [[3](#_ENREF_3)] for each insert size. The shortest transcript used for each simulation was 100nt longer than the insert size to ensure good coverage for each transcript. We aligned all simulated reads to whole genome (same as step S01 of SOAPfuse). Based on the alignment results, we used our method as describe above to evaluate the insert sizes (Supplementary Note Table 1). SOAPfuse precisely assessed each insert size with only about 0.6% shifting from the expected, except the 100nt insert size.

**Supplementary Note Table 1. Insert size evaluation of simulated reads**

| **Simulated insert size (nt)** | **Evaluated insert size (nt)** | **Shifting from expected** | **Standard Deviation of observed insert sizes** |
| --- | --- | --- | --- |
| 100 | 105 | 5.05% | 16.34 |
| 200 | 201 | 0.67% | 52.38 |
| 300 | 301 | 0.50% | 50.92 |
| 400 | 402 | 0.62% | 76.09 |
| 500 | 503 | 0.66% | 83.27 |
| 600 | 602 | 0.28% | 47.36 |
| 700 | 703 | 0.42% | 66.04 |
| 800 | 803 | 0.32% | 58.19 |
| 900 | 903 | 0.36% | 71.27 |
| 1,000 | 1,004 | 0.37% | 79.66 |

## Trimming and realigning the reads

Now the latest protocols for NGS RNA-Seq library preparation can generate paired-end reads with an insert size shorter than the total length of both reads (with the 3' ends of both reads overlapped). The paired-end reads with overlapped 3' ends may come from the junction regions containing the junction sites and these paired-end reads are not mapped to the reference if the overlapped regions cover the junction sites. These reads are components of FUM generated in step S02 (Figure S2) and cannot become span-reads, which will reduce the capability of fusion detection. SOAPfuse estimates whether the number of these paired-end reads with overlapped 3' ends exceeds the threshold (20% of total reads in default). If yes, or the user enables a trimming operation accessible in the configuration file, SOAPfuse will iteratively trim and realign FUM reads to annotated transcripts (Figure 7 and step S03 in Figure S2). The length of reads after trimming should be at least 30 nts (default parameter in SOAPfuse). The trimmed reads that are able to be mapped to annotated transcripts are stored in SE-S03. Two steps were used to finish the trimming and realigning operation: 1) FUM Reads were progressively trimmed off 5 bases from the 3'-end and mapped to annotated transcripts again until a match was found. 2) Using the same strategy, we trimmed the remaining FUM reads from the 5'-end. All mapped paired-end reads from above two steps were merged together (step S04 in Figure S2).

## Identifying candidate gene pairs

From all discordant aligned reads, SOAPfuse seeks span-reads to support candidate gene pairs (step S05 in Figure S2). Both the span-reads that mapped uniquely to reference and the trimmed reads that have multiple-hits were used to detect the candidate gene pairs. The maximum hits for each span-read is a parameter in the configuration file. To insure accurate detection of the fusion gene pairs, SOAPfuse imposes several filtrations on the predicted candidate gene pairs as follows:

1. Gene pairs from the same gene families are filtered out because these gene pairs always have similar sequences with each other that may mislead to spurious fusions.
2. Gene pairs that overlap with each other are eliminated (see Figure 6b in the main text).
3. For a given gene pair, gene A and B, there are two candidate fusion events with opposed up- and down-stream genes: *5'-A-B-3'* and *5'-B-A-3'*. We excluded the fusion events that are supported with less than 40% (the default parameter in the configuration file of SOAPfuse) of total spans-reads for the gene pairs.

## Determining the upstream and downstream genes in the fusion events

After obtaining the candidate gene pairs, the upstream and the downstream genes of the fusion were determined based on the information from span-read alignment against the reference. In the process of paired-end sequencing, the fragments are sequenced from bilateral edges to the middle part: one end starts from 3' end of the fragment, while the other end starts from 3' end of the complementary base-pairing sequence of the fragment (Figure 8a in the main text). This information is used to define the up- and down-stream genes in a fusion transcript.

A span-read (paired-end reads 'a' and 'b') supports candidate gene pair (*Gene A* and *Gene B*). According to the serial number ('1' or '2') and mapped orientation ('+' or '-') of paired-end reads (read 'a' and 'b'), there are 16 combinations, but only four are rational. These four combinations support two types of fusions in which the upstream and downstream genes are different (see Table 3 in the main text). The judgment rule is: the gene aligned by read in the plus orientation must be the upstream gene. Here, we presume that read 'a' maps to *Gene A* and read 'b' maps to *Gene B* (Figure 8b-c in the main text). In Figure 8b of the main text, read 'a' aligns to *Gene A* (annotated transcripts) in the plus orientation, so *Gene A* must be the upstream gene; while in Figure 8c of the main text, read 'b' aligns to *Gene B* in the plus orientation, so *Gene B* must be the upstream gene. According to this rule, SOAPfuse defines the upstream and downstream genes in fusion events.

## Getting the non-redundant transcript from multiple transcripts of the gene

Generally, lots of genes have more than one transcript due to the alternative splicing. To simplify the detection of junction sites, we integrated the different transcripts from a given gene to get a non-redundant transcript sequence (see Supplementary Note Figure 1), which was used to detect the fusion events in SOAPfuse method.


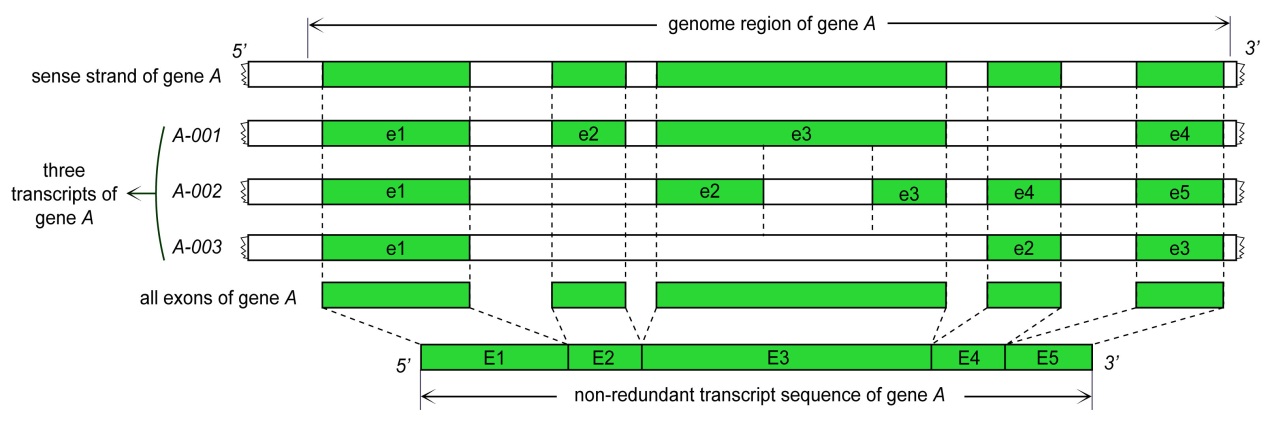


**Supplementary Note Figure 1:** Model of non-redundant transcript sequence from the gene A. Exons of gene A are in green. Gene A has three transcripts: A-001, A-002 and A-003.

## Getting the fused regions

Two methods were used to define the fused regions in gene pairs which contain the junction sites. In the first method, SOAPfuse bisects each FUM read, and generates two isometric segments, each called as half-unmapped read (HUM read; step S06 in Figure S2). HUM reads are aligned against candidate gene pairs with SOAP2. A genuine junction read (junc-read) should have at least one HUM read which does not cover the junction site and could map to one of the paired genes. Based on the mapped HUM read, SOAPfuse extends one HUM read-length from the mapped position in non-redundant transcripts to define the fused region wherein the junction site might be located (Figure 9a in the main text). For HUM reads with multiple-hits, all locations of the hits are taken into account. Original reads of mapped HUM reads are called as useful-unmapped reads (UUM read).

SOAPfuse also uses span-reads to detect the fused regions in candidate gene pairs (step S07-a in Figure S2). Span-reads, the paired-end reads supporting the candidate fusion gene pairs, are derived from the fused transcripts and the junction sites are often located in regions of the fused transcripts between the both ends of span-reads. For upstream and downstream genes, we can extend one region with length equal to insert size (evaluated in step S01) from the mapped position of each 3'-end span-read to estimate the fused region covering the junction site (Figure 9b in the main text). Every gene pair is always supported at least two span-reads, corresponding to several fused regions that may have overlaps with each other. We presumed that end 1 of a span-read mapped to position MP1 in Gene A, and end 2 of the span-read mapped to position MP2 in Gene B. The length of end 1 and 2 of span-read is RL1 and RL2 respectively. The average of insert sizes (INS) and their standard deviation (SD) were evaluated in step S01. The fused regions were estimated by the following intervals:

The intervals of fused regions for the upstream genes


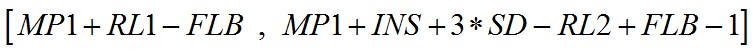


And the intervals of fused regions for the downstream genes


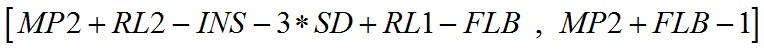


In above formula, a flanking region with length of FLB was considered because sometimes a few bases from the 3'-end of a span-read cover the junction sites in the mismatch-allowed alignment.

SOAPfuse combined the fused regions determined by above two methods to detect the junction sites using the partial exhaustion algorithm as described below.

## Construction of fusion junction sequences library with partial exhaustion algorithm

To simplify the explanation of the algorithm, we called the fused regions determined by above two methods as fused regions 1 and fused regions 2, respectively. Fused region 1, defined by the mapped HUM reads, is a small region covering the junction sites with length smaller than one NGS read. Fused region 2 is a large region defined by the NGS library insert sizes, which are always much longer than HUM reads. Generally, fused region 1 is more useful than fused region 2 to define the junction sites.

However, not all mapped HUM reads are from genuine junc-reads. Sometimes, one unmapped read from a given gene do not mapped this gene just due to more mismatches than allowed amount by SOAP2. The unmapped reads like this are not junc-reads and after the bisection into two HUM reads, one of the HUM reads could be mapped to the original gene, which result in spurious fused regions. Fused region 2 involves alignments of two ends of span-read simultaneously, which are also filtered by several effective criteria ("obtaining candidate gene pairs" section). SOAPfuse combined the fused regions 1 and 2 to efficiently define the junction sites. SOAPfuse classifies fused region 2 into two types of sub-regions: overlapped parts between fused regions 1 and 2 are called as credible-region, while rest parts of fused region 2 are called as potential-region (Figure 10a in the main text).

In order to build the fusion junction sequences library, we covered the fused region 2 from each of gene pairs with ‘tiles’ that are spaced 1 nt apart and finally we generate the candidate fusion junction library by creating all pair-wise connections between these tiles (Figure 10b in the main text). To eliminate the false positives in the junction sequences library, only the junction sequences in which at least one of the two junction sites in a gene pair is located in the credible-region were selected for further analysis. SOAPfuse carried out this partial exhaustion algorithm to reduce the size of the putative junction library and retain genuine junction sequences as much as possible.

## Detection of junction sites in fusion transcripts

To identify the junction sites of fusion events, we mapped the useful-unmapped-reads (UUM reads, see section "Getting the fused regions") to the putative fusion junction sequences library (step S07-b in Figure S2). We required that a candidate fusion should be supported by multiple junction reads that spanned the junction of the two genes with at least 5bps (a default parameter in the configuration file) (step S08 in Figure S2). In addition, the mismatches in the 5bps regions at both sides of junction sites should not exceed the threshold (0 as default) when the junction reads were aligned against the junction sequences. Furthermore, the counterpart end of the real junction reads (junction read is one of the both ends in the paired-end read) must map to one gene of the gene pair, or should be junc-read supporting the same fusion event. Using above strategy, SOAPfuse detected the putative fusion transcripts. Then, we carried out several methods to exclude false positives (Figure 6c and step S09 in Figure S2). Some of the neighboring genes represent a shared chromosomal region, and reads aligned to overlapping regions may also create artificial fusions. Therefore, SOAPfuse removes the neighboring gene pairs that have overlapping regions. Additionally, since gene pairs that contain highly similar sequences may cause ambiguous alignments, SOAPfuse also filters out the fusion transcripts in which the junction sites locate in the similar sequences between the gene pairs. After above analysis, SOAPfuse reports high-confident fusion transcripts and also provides the predicted junction sequences for further RT-PCR experimental validations. SVG figures are also created, showing the alignments of supporting reads on junction sequences and expression level of gene pairs (e.g., Additional file 11, Figure S3).

## Classification of fusion transcripts

SOAPfuse classifies the detected fusion transcripts into five sub-types as follows:

1. Fusion transcripts arising from the inter-chromosomal genes with different DNA strands (INTERCHR-DS for short). This type of fusion transcripts may be caused by the inter-chromosomal inversion.
2. Fusion transcripts arising from the inter-chromosomal genes with same DNA strand (INTERCHR-SS for short). This type of fusion may be caused by the inter-chromosomal translocation.
3. Fusion transcripts arising from the intra-chromosomal genes with different DNA strands (INTRACHR-DS for short). This type of fusion may be caused by the intra-chromosomal inversion.
4. This type of fusion transcripts arise from the intra-chromosomal genes with same DNA and the upstream and downstream genes in the events are reverse to their genomic coordinates (in other words, the upstream genes in the events are at downstream genomic locus of the downstream genes of the fusion events)(INTRACHR-SS-RGO for short). This type of fusion may be caused by the intra-chromosomal translocation.
5. This type of fusion transcripts arise from the intra-chromosomal genes with same DNA strand and the upstream and downstream genes in the fusion events are consistent with their genomic coordinates (INTRACHR-SS-OGO-xxGAP for short, in which the ‘xx’ indicates the number of other genes in the regions between the gene pairs). According to the distance between the gene pairs, this type of fusion may be caused by intra-chromosomal translocation, deletion or read-through.

There are several mechanisms that generate the fusion transcripts, including trans-splicing [[4-7](#_ENREF_4)], read-through transcripts produced by adjacent genes [[8](#_ENREF_8)] and chimeric transcripts from the genome rearrangement. SOAPfuse is not able to distinguish the fusion transcripts created by the genome rearrangement from the ones from trans-splicing. The whole genome sequencing or PCR on DNA level can define the origin of the fusion transcripts.

# EVALUATION OF SOAPFUSE PERFORMANCE

## Introduction

To assess the performance of SOAPfuse, We compared SOAPfuse with other five tools (Additional file 2, Table S2) on three RNA-Seq datasets. The first dataset includes two previous published cancer studies. They confirmed some fusions transcripts and provided Sanger sequences of validated fusions. The second one is a simulated RNA-Seq dataset, which contains 150 fusions simulated based on human annotated transcripts (Ensembl release 59th annotation database [[9](#_ENREF_9), [10](#_ENREF_10)]). The third one is RNA-Seq data from two bladder cancer cell lines we provided.

We ran all tools to analyze each dataset. Based on first dataset, we compared sensitivity of detection of known fusions and computing resources (CPU time and memory usage). On second dataset, false negative (FN) and false positive (FP) rates were compared. For the third dataset, we carried out experimental validations for fusion transcripts detected by SOAPfuse.

## The reasons for abandoning FusionSeq and FusionMap in comparison

We initially included FusionSeq [[11](#_ENREF_11)] and FusionMap [[12](#_ENREF_12)] among the tools for performance evaluation, but finally abandoned both methods due to computational limitations. We tried to run FusionSeq on the three datasets mentioned above, but during this work we found that it generated lots of temporary files which cost almost 1TB storage per sample. So, we gave up FusionSeq because of computing resource limitation. FusionMap is designed based on the Windows system and it also runs successfully in the Linux environment with the help of virtual machine. However, it is not suitable for analyzing large amount of RNA-Seq data. Therefore, we also gave up the FusionMap in our evaluation work.

## Criterion for detecting the known fusion events

To evaluate the performance and sensitivity of fusion detection, we applied these tools to the first and the second datasets mentioned above, in which the junction sites of fusion transcripts had been defined. All tools were run on the same release (hg19) of human reference genome sequence. We considered the known fusions were re-discovered by the tools if the distance between the junction sites detected by tools and the real sites is smaller than 10 bps based on the genome sequence or transcript sequences.

## Released RNA-Seq data in the first published dataset

The first dataset includes RNA-Seq data from two previous studies: (i) The study of six melanoma samples and one chronic myelogenous leukemia (CML) sample, in which 15 fusion transcripts were confirmed [[13](#_ENREF_13)]; and (ii) The research of breast cancer cell lines, reporting 27 confirmed fusions [[14](#_ENREF_14)]. We downloaded the RNA-Seq data from NCBI Sequence Read Archive (SRA). See Table S1 in Additional file 1 for detailed information on all confirmed fusions.

## Software Parameters used for two previously published studies

For the first dataset released by two previous cancer studies, we set standard as low as possible to detect more fusion transcripts. Several tools were tried with different parameters for many times to re-discover the most known fusion events with the shortest CPU time, especially for deFuse [[15](#_ENREF_15)], which were adjusted several times for its frequent nonzero-errors. However, we found that maximum memory usage is not significantly associated with parameters. The final parameters are as follows.

### Parameters for RNA-Seq data from the melanoma research

For SOAPfuse,

Using BWA [[2](#_ENREF_2)] to filter out unmapped reads caused by small indels (set '**PA_s02_realign**' as 'yes' in the configuration file); using the credible regions supported by 60% span-reads for the partial exhaustion calculation (Set '**PA_s07_the_min_cons_for_credible_fuse_region**' as 0.6); only seeking candidate gene pairs supported by at least 3 span-reads (set '**PA_s07_the_minimum_pe_support_reads**' as 3).

For TopHat-Fusion tophat step, we used the following flags:

**--allow-indels** **--no-coverage-search** **--fusion-min-dist** 30000 **--fusion-anchor-length** 10

There are two insert sizes: 500nt and 350nt. Read-length is 51nt (paired-end). For 500nt, we used flags: **-r** 398 **--mate-std-dev** 200; and for 350nt, we used: **-r** 248 **--mate-std-dev** 150.

For TopHat-Fusion tophat-fusion-post step, we used the following flags:

**--num-fusion-reads** 1 **--num-fusion-pairs** 1 **--num-fusion-both** 1

For deFuse,

Two parameters ('**span_count_threshold**' and '**split_count_threshold**', [number/number] for short in the Supplementary Note) are related to the filtering criteria. For example, all the known fusion transcripts were detected by deFuse if [5/3] was used for the samples M990802, M980409 and K-562. For the samples M000216, M010403, 501-MEL and M000921, we tried [1/1], [1/2] and [2/3], but deFuse failed to run and returned a ‘nonzero warning’. After trying many times, we finally used [2/2] for the samples M000216 and M000921, and [5/3] for the samples M990802, M980409, M010403, 501-MEL and K-562.

For FusionHunter,

In the configuration file, we set '**segment_size**' as half of read-length; '**PAIRNUM**' as 2; '**MINSPAN**' as 1; and '**MINOVLP**' as 8.

For chimerascan, we used the following flags:

**-v** **--quals** sanger **--processors** 4 **--anchor-min** 5 **--filter-unique-frags** 1

For SnowShoes-FTD,

In the configuration file, we set '**$distance**' as 5000; '**$minimal**' as 1; and '**$max_fusion_isoform**' as 5.

### Parameters for RNA-Seq data from the breast cancer research

For SOAPfuse,

Gene symbols which contain dot characters were included for detection of fusion transcripts (set '**PA_s05_save_genes_name_with_dot**' as 'yes' in the configuration file); we used the credible regions supported by 60% span-reads for the partial exhaustion calculation (Set '**PA_s07_the_min_cons_for_credible_fuse_region**' as 0.6); we sought candidate gene pairs supported by at least 3 span-reads (set '**PA_s07_the_minimum_pe_support_reads**' as 3).

For TopHat-Fusion tophat step, we used the following flags:

**--allow-indels** **--no-coverage-search** **--fusion-min-dist** 30000 **--fusion-anchor-length** 10

We also used '**–r** 50 **--mate-std-dev** 80' for the samples BT-474 and SK-BR-3; we used '**-r** 0 **--mate-std-dev** 80' for samples MCF-7 and KPL-4.

For TopHat-Fusion tophat-fusion-post step, we used the following flags:

**--num-fusion-reads** 1 **--num-fusion-pairs** 1 **--num-fusion-both** 1

For deFuse,

For sample SK-BR-3, setting [5/3] could recover all known fusions. For samples BT-474, KPL-4 and MCF-7, we tried [1/1], [1/2] and [2/2], but failed with a nonzero warning. We finally confirmed [2/3] for samples KPL-4 and MCF-7; and [5/3] for samples BT-474 and SK-BR-3.

For FusionHunter,

In the configuration file, we set '**segment_size**' as half of read-length; '**PAIRNUM**' as 2; '**MINSPAN**' as 1; and '**MINOVLP**' as 8.

For chimerascan, we used the following flags:

**-v** **--quals** sanger **--processors** 4 **--anchor-min** 5 **--filter-unique-frags** 1

For SnowShoes-FTD,

In the configuration file, we set '**$distance**' as 5000; '**$minimal**' as 1; and '**$max_fusion_isoform**' as 5.

## Fusion transcripts missed by SOAPfuse in the breast cancer data

For the first dataset, SOAPfuse only missed one fusion event, *NFS1-PREX* in sample SK-BR-3 from RNA-Seq data of breast cancer and this fusion transcript was not detected by any other tools. SOAPfuse didn’t find any span-read which supported the gene pair, *NFS1* and *PREX.*

## Simulating the fusion transcripts

We simulated fusion transcripts by two steps. The first step is to select candidate gene pairs for simulation. And the second step is to simulate the fusion transcripts from the selected gene pairs. All work was done based on Ensembl release 59th annotation database.

### The first step of fusions simulation

In the first step of simulation experiment, we randomly selected any two genes from the human genome as the candidate gene pairs and filtered out the un-reasonable pairs as follows: (1) The distance between the paired genes in the same chromosome with same strand is less than 100kps (2) the gene pairs from the same gene families; and (3) the gene pairs in the blacklist provided by FusionMap [[12](#_ENREF_12)]. The three criteria were explained as follows:

Some methods filtered out the read-through transcripts from output of the software while other methods may not. Considering that there might be special and different filters on read-through transcripts in different methods, we avoided read-through fusions in the simulation work. To achieve this aim, the distance between the paired genes in the same chromosome with same strand should be at least 100kps (the 1st criterion for simulation).

Gene pairs that have high similar sequences may cause ambiguous read alignments, resulting in spurious fusion transcripts. So we required that each simulated gene pair should not be from the same gene family (the 2nd criterion for simulation).

In filtration work of FusionMap [[12](#_ENREF_12)], gene pairs from its blacklist were discarded. The gene blacklist includes mitochondrial and ribosomal genes according to Gene Ontology (GO), and pseudogenes according to three sources: Ensembl annotations, Entrez Gene Database and HUGO Gene Nomenclature Committee (HGNC). In our work, fusion candidates involving genes included in this blacklist will be removed (the 3rd criterion for simulation).

The remaining gene pairs were for the second step in the simulation work.

### The second step of fusions simulation

We randomly selected one transcript from each gene in every simulated gene pair and created the fused transcript by the paired transcripts. In our simulation work, the junction site in each transcript was random and the junction sites in the paired transcripts may locate at the exons edge (splicing junction) or in the middle of the exon regions. Furthermore, we required that the length of fused transcripts should be at least 500pbs, and that upstream and downstream sequences be longer than 100bps. After the simulation work, we obtained 150 fusion transcripts (Additional file 6, Table S6).

## Simulation of paired end RNA-Seq reads based on the simulated fusion transcripts

Based on the 150 simulated fusion transcripts, we used the short-read simulator provided by MAQ [[3](#_ENREF_3)] to generate paired end RNA-Seq reads (2 x 75nt; INS=160nt, SD=15). This yielded gradient sequencing depth (5-, 10-, 20-, 30-, 50-, 80-, 100-, 150- and 200- fold) for each fused transcripts to represent the different expression level of the transcripts. The detailed information on simulated RNA-Seq reads and the simulated supporting-reads for each fusion transcripts is in Table S5 and S7 of Additional file 6, respectively. The simulated RNA-Seq reads at each depth were mixed with background data (see section "Background data") to generate the final simulated RNA-Seq dataset.

## Background data

The background RNA-Seq data is from H1 human embryonic stem cells (hESCs) that were not expected to harbor any fusion transcripts. It was generated by the ENCODE Caltech RNA-Seq project [[16](#_ENREF_16), [17](#_ENREF_17)] and was also used as background by FusionMap [[12](#_ENREF_12)]. We downloaded it from NCBI Sequence Read Archive under accession number [SRR065491] and [SRR066679]. We filtered out the low quality reads and the remaining 19 million paired-end reads were mixed with the simulated reads generated as described in the above section to get the final simulated RNA-Seq dataset.

## Software parameters used for simulated RNA-Seq dataset

We divided the simulated dataset into two parts based on the expression levels of the fused transcripts: low levels (5~50-fold) and high levels (80~200-fold). And two different sets of software parameters were used for these two parts of the simulated dataset.

### Low standard parameters for low expression level of the fusion transcripts

For SOAPfuse,

The fusion transcripts should be supported by at least one span-read and one junc-read. If the both junction sites in the paired genes were in the middle of the exons, the fused transcripts should be supported by at least 2 span-reads and 2 junc-reads. In addition, the junc-reads should span the junction sites of paired genes with at least 5 bps. Furthermore, the genomic distance between paired genes with same strand in the same chromosome must be larger than 100,000bps.

For TopHat-Fusion tophat step, we used the following flags:

**-p** 8 **--allow-indels** **--no-coverage-search** **-r** 10 **--mate-std-dev** 100 **--fusion-min-dist** 90000 **--fusion-anchor-length** 10

For TopHat-Fusion tophat-fusion-post step, we used the following flags:

**--num-fusion-reads** 1 **--num-fusion-pairs** 1 **--num-fusion-both** 1

For deFuse,

We tested deFuse with low standard, but deFuse failed to run and returned 'nonzero return code'. Finally, we set **span_count_threshold** as 2; **split_count_threshold** as 2; and **dna_concordant_length** as 100,000.

### Strict software parameters for high expression levels of the fusion transcripts

For SOAPfuse,

The fusion transcripts should be supported by at least two span-read and two junc-read. In addition, the junc-reads should span the junction sites of paired genes with at least 10 bps. Furthermore, the genomic distance between paired genes with same strand in the same chromosome must be larger than 100,000bps.

For TopHat-Fusion tophat step, we used the following flags,

**-p** 8 **--allow-indels** **--no-coverage-search** **-r** 10 **--mate-std-dev** 100 **--fusion-min-dist** 90000 **--fusion-anchor-length** 10

For TopHat-Fusion tophat-fusion-post step, we used the following flags,

**--num-fusion-reads** 1 **--num-fusion-pairs** 1 **--num-fusion-both** 1

For deFuse,

We set **span_count_threshold** as 5, **split_count_threshold** as 3, **dna_concordant_length** as 100,000. We tried to test deFuse with lower standard, but deFuse could not detect true positive fusions any more.

## Calculation of the false negative (FN) and false positive (FP) rate

Among the six tools for evaluation, chimerascan [[18](#_ENREF_18)], FusionHunter [[19](#_ENREF_19)] and SnowShoes-FTD [[20](#_ENREF_20)] only detect events fused at the exon edge (splicing junction), illustrating their particular algorithms of searching for fusion transcripts with junction sites at the exon edge. There were about 70 simulated fusion transcripts with junction sites in the middle of the exons, so we abandoned these three tools and retained SOAPfuse, deFuse and TopHat-Fusion for the FN and FP rate evaluation. For each tool, we tried different parameters to make more simulated fusion transcripts detected. As a result, 149 (99%) of the 150 simulated events were detected, and 142 (94%) were identified by at least two tools, indicating our simulation work was reasonable. To be prudent, we calculated FN and FP rate for each tool based on these 142 events found by at least two algorithms.

For each tool, we independently calculated both the FN and FP rate at different sequencing depth. At a given depth, the number of the simulated fusion events detected by the tools was defined as the true positive (TP). The number of detected fusion transcripts that were not in the list of the simulated events (FP) was also assessed. Then, we calculated the FN and FP rate using the following formula:


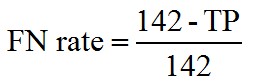


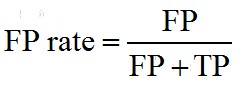


See the Table S8 in Additional file 7 for TP and FP of all tools and see the Table S9 in Additional file 7 for the simulated fusion events detected by the tools.

## Simulated fusion transcripts missed by SOAPfuse

Three fusion events, *STAMBP-RGPD1*, *IRAK1-XAGE2B* and *KHDRBS2-SYTL1*, were missed by SOAPfuse, but they were detected by both deFuse and TopHat-Fusion.

For *STAMBP-RGPD1* and *IRAK1-XAGE2B*, SOAPfuse reported fusions formed by their homogenous genes, *STAMBP-RGPD2* and *IRAK1-**XAGE2*, which were finally considered as false positives. Both of *XAGE2B* and *XAGE2* are in the chromosome X and have exactly same sequences*.* SOAPfuse detected the *IRAK1-XAGE2* instead *of IRAK1-XAGE2B* probably due to ambiguous reads alignment. The transcripts of *RGPD1* and *RGPD2* have the same sequences. Interestingly, there are two exons in *RGPD1* that merged to a single exon in *RGPD2*. We suspected that *RGPD2* are from the mechanism of retrotransposons. When the reads from *RGPD1* transcript were aligned against the whole genome sequence, they were more likely to map to the *RGPD2*, resulting in the *STAMBP-RGPD2* detected by SOAPfuse.

Although *KHDRBS2-SYTL1* was initially detected as the candidate gene pair by SOAPfuse, we did not detect the junction site in gene *SYTL1*. Detailed analysis showed that there are 8 different transcripts for the gene *SYTL1*, and SYTL1-007, the second shortest transcript, was selected in the simulation work. As Supplementary Note Figure 2 shown, the real fused region consists of sequences from exon 1 and exon 2 in SYTL1-007. To detect the junction site in the non-redundant transcript of *SYTL1,* SOAPfuse extended a region with length equal to insert size from the mapped position of one end of span-reads. However, SOAPfuse failed to detect the genuine fused region because there were 206bps region between exon 1 and 2 of SYTL1-007 and this intron region is annotated as exon region in other two transcripts of *SYTL1* and also in the non-redundant transcript of *SYTL1*.


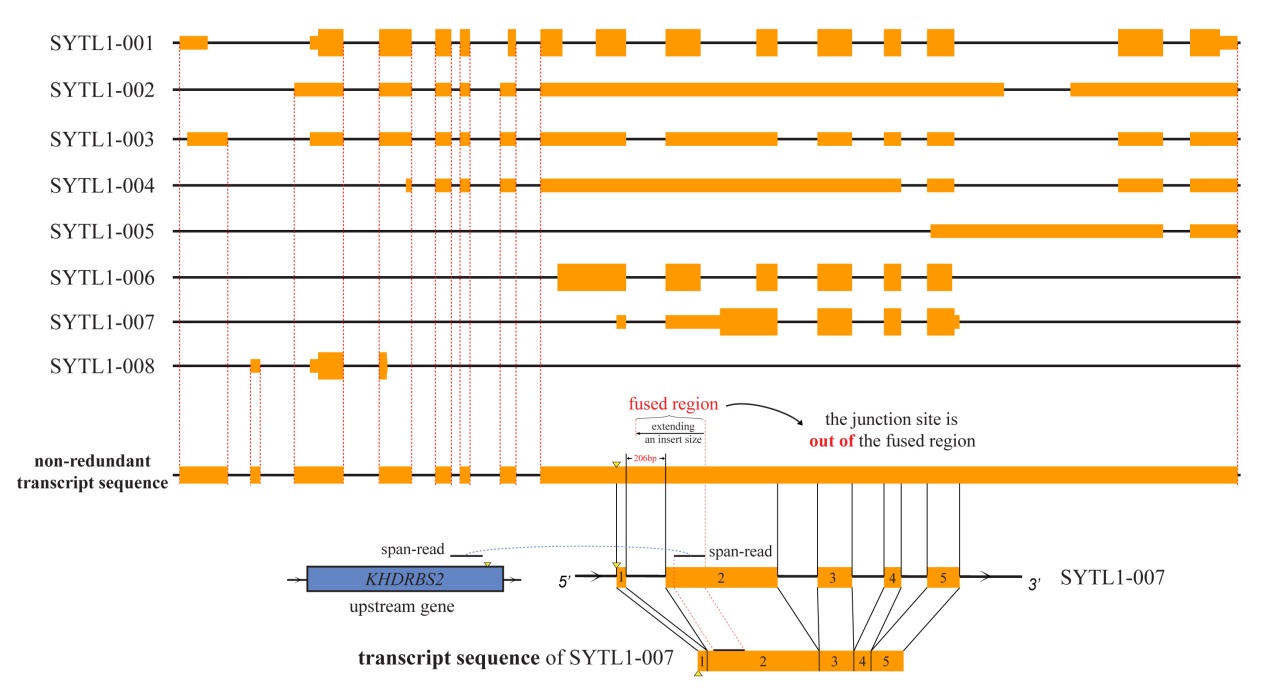


**Supplementary Note Figure 2:** non-redundant transcript sequence of *SYTL1*. SOAPfuse failed to detect the genuine fused region of SYTL1-007.

## Preliminary solutions to simulated events missed by SOAPfuse

The analysis of the three simulated events missed by SOAPfuse suggests that the SOAPfuse has difficulty in analyzing genes that have high similar sequences with other genes, and fusions involving short transcripts of the long genes. We have achieved some preliminary solutions to these shortcomings of SOAPfuse.

We re-aligned the paired-end reads from SE-S01, which were generated by alignment against whole genome sequence (step S01), to the annotated transcript sequences. This analysis aimed at retrieving the reads that ambiguously mapped to the homologous genes in the process of reads alignment against whole genome. Fusions *STAMBP-RGPD1* and *IRAK1-XAGE2B*, missed by SOAPfuse, were successfully detected by this strategy.

We then updated the whole algorithm from treating non-redundant transcripts to treating a single transcript. By this, SOAPfuse could detect fusions arising from the short transcripts of the long genes. Based on the new algorithm, the remaining missed event, *KHDRBS2-SYTL1*, was detected successfully.

Next, we will include these solutions in the future versions of SOAPfuse, and release them in official website as soon as possible.

## Software parameters used for bladder cancer cell line dataset

For RNA-Seq data from two bladder cancer cell lines, conservative parameter settings were used for SOAPfuse and deFuse. DeFuse used the default parameters. For SOAPfuse, the fusion events with junction sites at exon edge were required to be supported by at least 2 span-reads and 2 junc-reads, while events with junction sites in the middle of exons should be supported by at least 4 span-reads and 4 junc-reads. Then we tested the parameters used in deFuse to filter out the potential false positives from the result of SOAPfuse.

## Selecting predicted fusion transcripts to validate by experiment RT-PCR

SOAPfuse detected 16 fusion transcripts in the two bladder cancer cell lines. All 16 events were chosen for validation and 15 were confirmed by RT-PCR followed by Sanger sequencing. DeFuse initially identified 50 fusion transcripts in two cell lines. To fairly compare the SOAPfuse and deFuse, we also filtered out potential false positives by the strategies which were also used in the SOAPfuse and the remaining 10 events were selected for experiment validation: we excluded the fusion transcripts detected by deFuse if the events generated by gene pairs as follows:

1. Gene pairs that do not exist in Ensembl release 59th annotation database used in the SOAPfuse.
2. Fusion transcripts whose junction sites locate in the similar regions between the predicted gene pairs.
3. Gene pairs from the same gene families.
4. Fusion transcripts belonging to type of INTRACHR-SS-OGO-xxGAP (see section "classification of fusion transcripts") and in which the distance between is smaller than 20,000nt.

# WEBSITE

## Official Website

SOAPfuse belongs to the Short Oligonucleotide Analysis Package (SOAP) developed by BGI. SOAP has its official website, and all the sub-tools are available on it, including SOAPfuse (http://soap.genomics.org.cn/soapfuse.html).

Latest version, databases and config template of SOAPfuse can be downloaded from official website. Tutorial is displayed on the web-page, including installation, preparation before running, how to run SOAPfuse and explanation of the output. Work on the performance evaluation is also shown. We provide the configuration file (or the parameters list) and the result of each tool in the performance evaluation work. And for the simulated dataset, we also provide simulated RNA-Seq data (FASTQ format) in compressed package.

# REFERENCES

1. Li R, Yu C, Li Y, Lam TW, Yiu SM, Kristiansen K, Wang J: **SOAP2: an improved ultrafast tool for short read alignment.** *Bioinformatics* 2009, **25:**1966-1967.

2. Li H, Durbin R: **Fast and accurate short read alignment with Burrows-Wheeler transform.** *Bioinformatics* 2009, **25:**1754-1760.

3. Li H, Ruan J, Durbin R: **Mapping short DNA sequencing reads and calling variants using mapping quality scores.** *Genome research* 2008, **18:**1851-1858.

4. Sutton RE, Boothroyd JC: **Evidence for trans splicing in trypanosomes.** *Cell* 1986, **47:**527-535.

5. Krause M, Hirsh D: **A trans-spliced leader sequence on actin mRNA in C. elegans.** *Cell* 1987, **49:**753-761.

6. Rajkovic A, Davis RE, Simonsen JN, Rottman FM: **A spliced leader is present on a subset of mRNAs from the human parasite Schistosoma mansoni.** *Proceedings of the National Academy of Sciences of the United States of America* 1990, **87:**8879-8883.

7. Horiuchi T, Aigaki T: **Alternative trans-splicing: a novel mode of pre-mRNA processing.** *Biol Cell* 2006, **98:**135-140.

8. Akiva P, Toporik A, Edelheit S, Peretz Y, Diber A, Shemesh R, Novik A, Sorek R: **Transcription-mediated gene fusion in the human genome.** *Genome research* 2006, **16:**30-36.

9. Flicek P, Amode MR, Barrell D, Beal K, Brent S, Chen Y, Clapham P, Coates G, Fairley S, Fitzgerald S, Gordon L, Hendrix M, Hourlier T, Johnson N, Kahari A, Keefe D, Keenan S, Kinsella R, Kokocinski F, Kulesha E, Larsson P, Longden I, McLaren W, Overduin B, Pritchard B, Riat HS, Rios D, Ritchie GR, Ruffier M, Schuster M, et al: **Ensembl 2011.** *Nucleic acids research* 2011, **39:**D800-806.

10. Hubbard T, Barker D, Birney E, Cameron G, Chen Y, Clark L, Cox T, Cuff J, Curwen V, Down T, Durbin R, Eyras E, Gilbert J, Hammond M, Huminiecki L, Kasprzyk A, Lehvaslaiho H, Lijnzaad P, Melsopp C, Mongin E, Pettett R, Pocock M, Potter S, Rust A, Schmidt E, Searle S, Slater G, Smith J, Spooner W, Stabenau A, et al: **The Ensembl genome database project.** *Nucleic acids research* 2002, **30:**38-41.

11. Sboner A, Habegger L, Pflueger D, Terry S, Chen DZ, Rozowsky JS, Tewari AK, Kitabayashi N, Moss BJ, Chee MS, Demichelis F, Rubin MA, Gerstein MB: **FusionSeq: a modular framework for finding gene fusions by analyzing paired-end RNA-sequencing data.** *Genome biology* 2010, **11:**R104.

12. Ge H, Liu K, Juan T, Fang F, Newman M, Hoeck W: **FusionMap: detecting fusion genes from next-generation sequencing data at base-pair resolution.** *Bioinformatics* 2011, **27:**1922-1928.

13. Berger MF, Levin JZ, Vijayendran K, Sivachenko A, Adiconis X, Maguire J, Johnson LA, Robinson J, Verhaak RG, Sougnez C, Onofrio RC, Ziaugra L, Cibulskis K, Laine E, Barretina J, Winckler W, Fisher DE, Getz G, Meyerson M, Jaffe DB, Gabriel SB, Lander ES, Dummer R, Gnirke A, Nusbaum C, Garraway LA: **Integrative analysis of the melanoma transcriptome.** *Genome research* 2010, **20:**413-427.

14. Edgren H, Murumagi A, Kangaspeska S, Nicorici D, Hongisto V, Kleivi K, Rye IH, Nyberg S, Wolf M, Borresen-Dale AL, Kallioniemi O: **Identification of fusion genes in breast cancer by paired-end RNA-sequencing.** *Genome biology* 2011, **12:**R6.

15. McPherson A, Hormozdiari F, Zayed A, Giuliany R, Ha G, Sun MG, Griffith M, Heravi Moussavi A, Senz J, Melnyk N, Pacheco M, Marra MA, Hirst M, Nielsen TO, Sahinalp SC, Huntsman D, Shah SP: **deFuse: an algorithm for gene fusion discovery in tumor RNA-Seq data.** *PLoS computational biology* 2011, **7:**e1001138.

16. Birney E, Stamatoyannopoulos JA, Dutta A, Guigo R, Gingeras TR, Margulies EH, Weng Z, Snyder M, Dermitzakis ET, Thurman RE, Kuehn MS, Taylor CM, Neph S, Koch CM, Asthana S, Malhotra A, Adzhubei I, Greenbaum JA, Andrews RM, Flicek P, Boyle PJ, Cao H, Carter NP, Clelland GK, Davis S, Day N, Dhami P, Dillon SC, Dorschner MO, Fiegler H, et al: **Identification and analysis of functional elements in 1% of the human genome by the ENCODE pilot project.** *Nature* 2007, **447:**799-816.

17. Raney BJ, Cline MS, Rosenbloom KR, Dreszer TR, Learned K, Barber GP, Meyer LR, Sloan CA, Malladi VS, Roskin KM, Suh BB, Hinrichs AS, Clawson H, Zweig AS, Kirkup V, Fujita PA, Rhead B, Smith KE, Pohl A, Kuhn RM, Karolchik D, Haussler D, Kent WJ: **ENCODE whole-genome data in the UCSC genome browser (2011 update).** *Nucleic acids research* 2011, **39:**D871-875.

18. Iyer MK, Chinnaiyan AM, Maher CA: **ChimeraScan: a tool for identifying chimeric transcription in sequencing data.** *Bioinformatics* 2011, **27:**2903-2904.

19. Li Y, Chien J, Smith DI, Ma J: **FusionHunter: identifying fusion transcripts in cancer using paired-end RNA-seq.** *Bioinformatics* 2011, **27:**1708-1710.

20. Asmann YW, Hossain A, Necela BM, Middha S, Kalari KR, Sun Z, Chai HS, Williamson DW, Radisky D, Schroth GP, Kocher JP, Perez EA, Thompson EA: **A novel bioinformatics pipeline for identification and characterization of fusion transcripts in breast cancer and normal cell lines.** *Nucleic acids research* 2011, **39:**e100.
