# Supplementary material for: SOAPfuse: an algorithm for identifying fusion transcripts from paired-end RNA-Seq data
Source: Genome Biol. 2013 Feb 14;14(2):R12. doi: 10.1186/gb-2013-14-2-r12 (PMC4054009; doi:10.1186/gb-2013-14-2-r12)
Supplement: Additional file 9 — Figure S1 - models of fusion transcripts generated by genome rearrangement. (a) Fusion transcript created by genomic inversion of Gene A and Gene B, which are from different DNA strands. (b) Fusion transcript formed by genomic translocation in which Gene C and Gene D are from the same DNA strand and are far from each other. [file gb-2013-14-2-r12-S9.PDF]

Figure S1

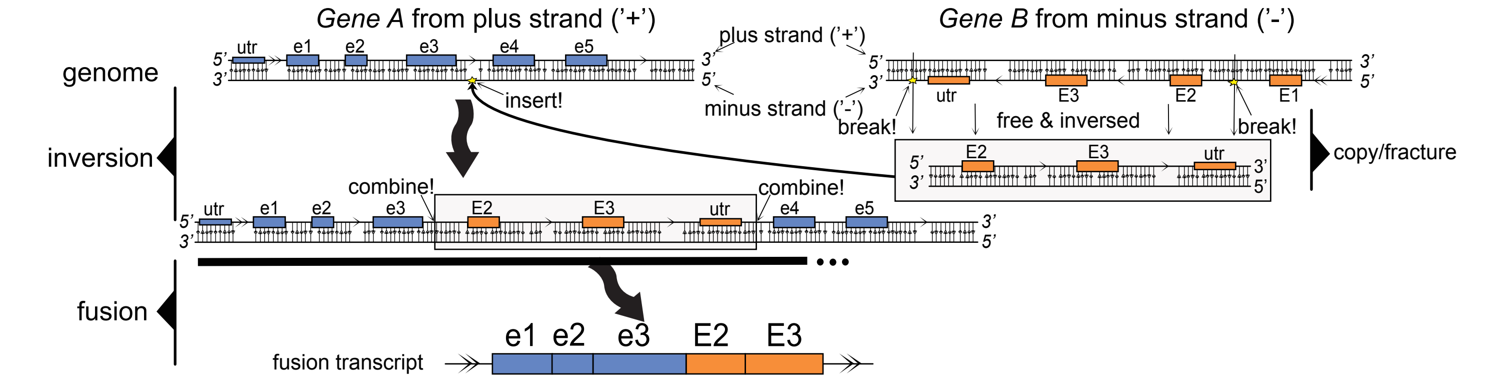

(a) fusion transcript created by genomic inversion

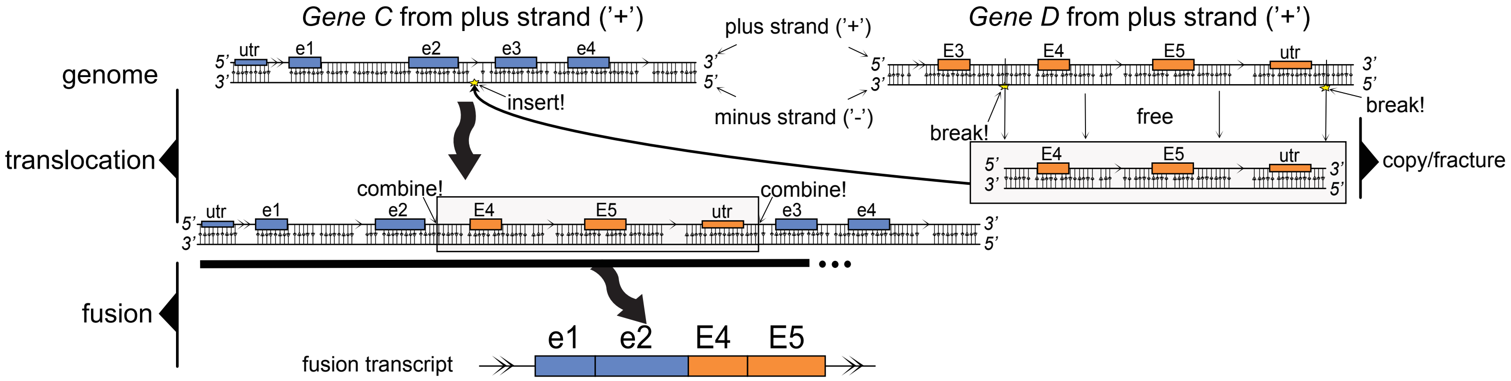

(b) fusion transcript formed by genomic translocation
