# Supplementary figures and images for: SOAPfuse: an algorithm for identifying fusion transcripts from paired-end RNA-Seq data
Source: Genome Biol. 2013 Feb 14;14(2):R12. doi: 10.1186/gb-2013-14-2-r12 (PMC4054009; doi:10.1186/gb-2013-14-2-r12)

Figure S2

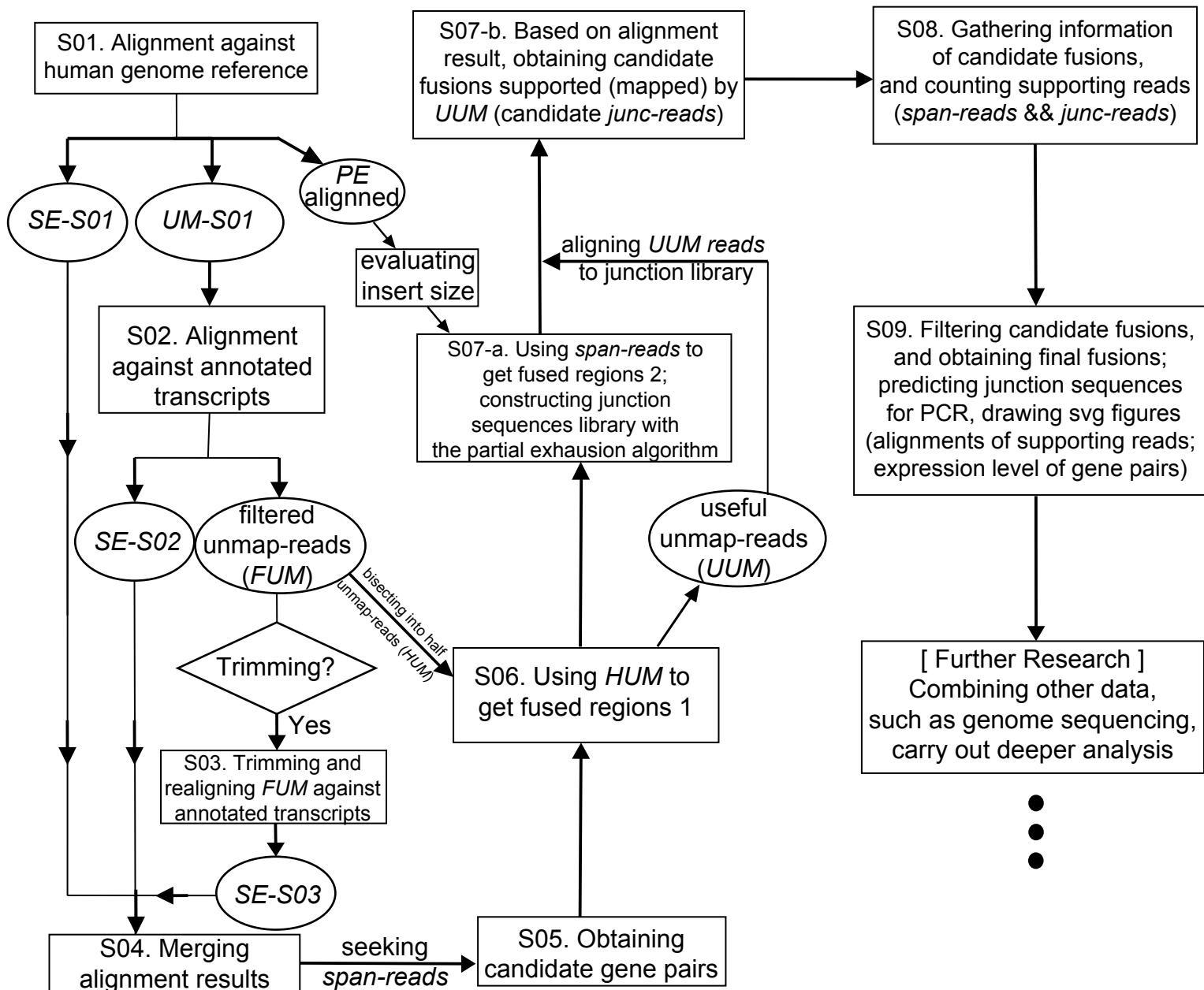

Supplement: Additional file 10 — Figure S2 - schematic diagrams of nine steps in the SOAPfuse pipeline. The SOAPfuse algorithm consists of nine steps (from S01 to S09) and details of each step are in the Materials and methods or Additional file 3. [file gb-2013-14-2-r12-S10.PDF]

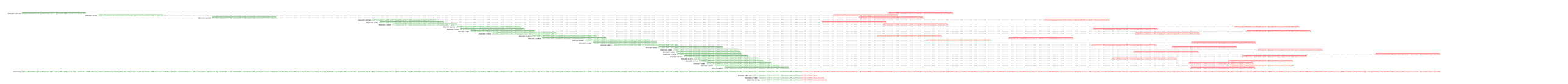

Supplement: Additional file 12 — Figure S3 - schematic diagrams of fusion event RECK-ALX3. (a) Alignment of supporting reads against the predicted junction sequence. The upstream part of the junction sequence is in green, and the downstream part is in red. Span-reads are displayed above the predicted junction sequence with the colored dotted line linking paired-end reads. Junc-reads are shown below the junction sequence. (b,c) Expression analysis of the exons in RECK and ALX3 by RNA-Seq read coverage. Transcripts of RECK and ALX3 are shown below the coordinates. The junction site is shown as a red round dot and a green arrow indicates the transcript orientation in the genome sequence. The region covered by the red line is the region mapped by supporting reads. In this case, we found that the expression levels of RECK and ALX3 exons at bilateral sides of junction sites are significantly different. The exons involved in the fusion transcript are expressed more highly than other ones. [file gb-2013-14-2-r12-S12.ZIP › Figure S3/Figure S3-a.png]

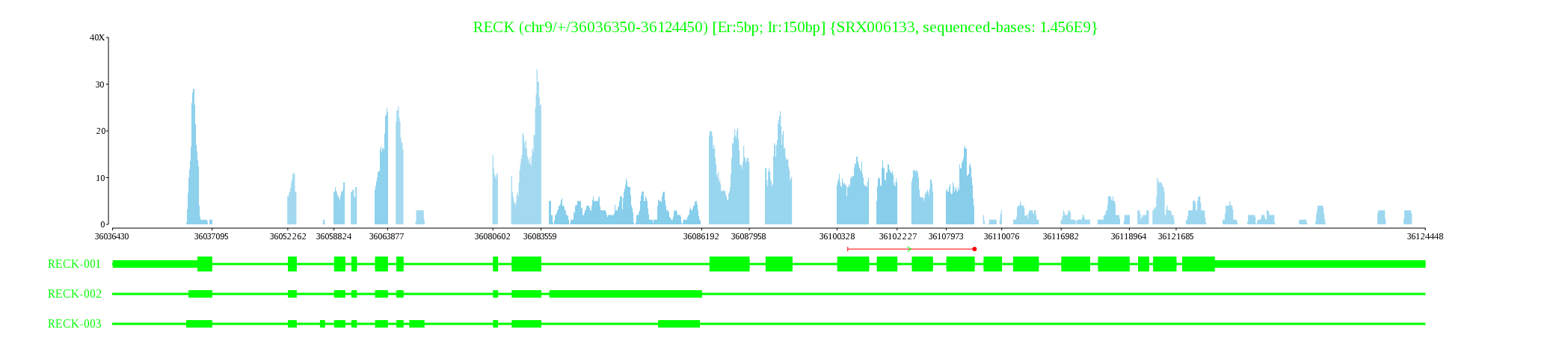

Supplement: Additional file 12 — Figure S3 - schematic diagrams of fusion event RECK-ALX3. (a) Alignment of supporting reads against the predicted junction sequence. The upstream part of the junction sequence is in green, and the downstream part is in red. Span-reads are displayed above the predicted junction sequence with the colored dotted line linking paired-end reads. Junc-reads are shown below the junction sequence. (b,c) Expression analysis of the exons in RECK and ALX3 by RNA-Seq read coverage. Transcripts of RECK and ALX3 are shown below the coordinates. The junction site is shown as a red round dot and a green arrow indicates the transcript orientation in the genome sequence. The region covered by the red line is the region mapped by supporting reads. In this case, we found that the expression levels of RECK and ALX3 exons at bilateral sides of junction sites are significantly different. The exons involved in the fusion transcript are expressed more highly than other ones. [file gb-2013-14-2-r12-S12.ZIP › Figure S3/Figure S3-b.png]

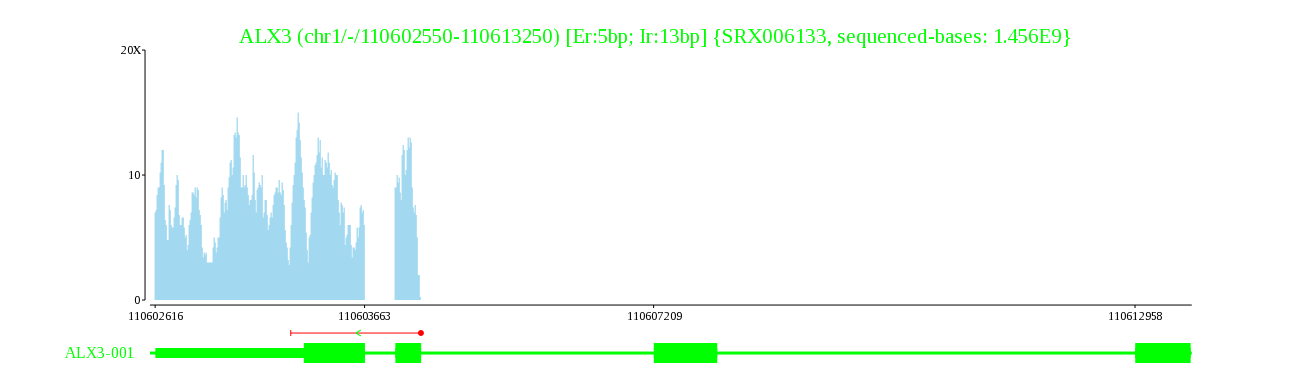

Supplement: Additional file 12 — Figure S3 - schematic diagrams of fusion event RECK-ALX3. (a) Alignment of supporting reads against the predicted junction sequence. The upstream part of the junction sequence is in green, and the downstream part is in red. Span-reads are displayed above the predicted junction sequence with the colored dotted line linking paired-end reads. Junc-reads are shown below the junction sequence. (b,c) Expression analysis of the exons in RECK and ALX3 by RNA-Seq read coverage. Transcripts of RECK and ALX3 are shown below the coordinates. The junction site is shown as a red round dot and a green arrow indicates the transcript orientation in the genome sequence. The region covered by the red line is the region mapped by supporting reads. In this case, we found that the expression levels of RECK and ALX3 exons at bilateral sides of junction sites are significantly different. The exons involved in the fusion transcript are expressed more highly than other ones. [file gb-2013-14-2-r12-S12.ZIP › Figure S3/Figure S3-c.png]
